# Characterization of somatic structural variations in 528 Chinese individuals with Esophageal squamous cell carcinoma

**DOI:** 10.1038/s41467-022-33994-3

**Published:** 2022-10-22

**Authors:** Heyang Cui, Yong Zhou, Fang Wang, Caixia Cheng, Weimin Zhang, Ruifang Sun, Ling Zhang, Yanghui Bi, Min Guo, Yan Zhou, Xinhui Wang, Jiaxin Ren, Ruibing Bai, Ning Ding, Chen Cheng, Longlong Wang, Xuehan Zhuang, Mingwei Gao, Yongjia Weng, Yueguang Wu, Huijuan Liu, Shuaicheng Li, Shubin Wang, Xiaolong Cheng, Yongping Cui, Zhihua Liu, Qimin Zhan

**Affiliations:** 1 Cancer Institute, Shenzhen Key Laboratory of Gastrointestinal Cancer Translational Research, Peking University Shenzhen Hospital, Shenzhen Peking University-the Hong Kong University of Science and Technology (PKU-HKUST) Medical Center; Institute of Cancer Research, Shenzhen Bay Laboratory, Shenzhen, 518028 China; 2 Key Laboratory of Cellular Physiology of the Ministry of Education, Department of Pathology, Shanxi Medical University, Taiyuan, Shanxi 030001 China; 3 City University of Hong Kong Shenzhen Research Institute, Shenzhen, China; 4 Department of Pathology, The First Hospital, Shanxi Medical University, Jinzhong, 030001 China; 5 Key laboratory of Carcinogenesis and Translational Research (Ministry of Education/Beijing), Laboratory of Molecular Oncology, Peking University Cancer Hospital & Institute, Beijing, 100142 China; 6 Research Unit of Molecular Cancer Research, Chinese Academy of Medical Sciences, Beijing, China; 7 Department of tumor biobank, Shanxi Provincial Cancer Hospital, Jinzhong, China; 8 Department of Oncology, Shenzhen Key Laboratory of Gastrointestinal Cancer Translational Research, Cancer Institute, Peking University Shenzhen Hospital, Shenzhen-Peking University-Hong Kong University of Science and Technology Medical Center, Shenzhen, 518036 China; 9 State Key Laboratory of Molecular Oncology, National Cancer Center/National Clinical Research Center for Cancer/Cancer Hospital, Chinese Academy of Medical Sciences and Peking Union Medical College, Beijing, 100021 China

**Keywords:** Cancer genomics, Genetics research, Oncogenes, DNA sequencing, Oesophageal cancer

## Abstract

Esophageal squamous cell carcinoma (ESCC) demonstrates high genome instability. Here, we analyze 528 whole genomes to investigate structural variations’ mechanisms and biological functions. SVs show multi-mode distributions in size, indicating distinct mutational processes. We develop a tool and define five types of complex rearrangements with templated insertions. We highlight a type of fold-back inversion, which is associated with poor outcomes. Distinct rearrangement signatures demonstrate variable genomic metrics such as replicating time, spatial proximity, and chromatin accessibility. Specifically, fold-back inversion tends to occur near the centrosome; TD-c2 (Tandem duplication-cluster2) is significantly enriched in chromatin-accessibility and early-replication region compared to other signatures. Analyses of TD-c2 signature reveal 9 TD hotspots, of which we identify a hotspot consisting of a super-enhancer of *PTHLH*. We confirm the oncogenic effect of the *PTHLH* gene and its interaction with enhancers through functional experiments. Finally, extrachromosomal circular DNAs (ecDNAs) are present in 14% of ESCCs and have strong selective advantages to driver genes.

## Abstract (web summary)

The biological and clinical implications of high genome instability in esophageal squamous cell carcinoma (ESCC) remain to be explored. Here, the authors analyse 528 whole genomes and investigate the landscape and molecular mechanisms underlying structural variations in ESCC patients.

## Introduction

Esophageal cancer is one of the most aggressive cancers and the sixth leading cause of cancer death worldwide^1^. More than 50% of patients occur in China, and esophageal squamous cell carcinoma (ESCC) is the main pathological type^2^. The five-year survival rate is approximately 30.3%^3^. Understanding the genetic basis and underlying mutational mechanisms is essential for drug development and clinical treatment. Over the past decade, researchers have characterized the genetic landscape of ESCC from various aspects, such as different populations, multi-regional sequencing, and omics-integration analyses^4–9^. Based on genome sequencing, these studies explored the driver mutations, mutational process, key pathways, or clonal dynamics during ESCC tumorigenesis. They found recurrently mutated genes such as *NOTCH1, ZNF750*, and *NFE2L2*, and mutational processes associated with drinking, and the efficiency of platinum-based therapy^8–11^. However, although few performed whole-genome sequencing^9,12^, the analyses regarding SVs and their mechanisms are still limited.

SVs are genomic rearrangements that lead to duplication, deletion, or inversion of genomic segments. In this study, we call an event involving two or more SVs a complex rearrangement. In contrast, a simple rearrangement involves only one SV. Each SV consists of two breakpoints, and is classified into five types: deletion-type(+/−), TD-type(−/+), head-to-head(+/+), tail-to-tail(−−) type, and translocation^13^. The deletions and TDs of different sizes seem to source from distinct mutational processes and display variable functional properties in human cancers^14^. Numerous complex rearrangements exist and often lead to high-level amplification of multiple oncogenes or disruption of tumor suppressors. For instance, ecDNA, BFB(Breakage-fusion-bridge), chromothripsis, and chromoplexy are prevalent in human cancer and associated with clinal outcome^15–18^. Recently, complex rearrangements with templated insertions were proposed, and they often consist of several templated copies from distinct genomic regions. Templated insertions recurrently activate *TERT* in liver cancer^14^. However, the patterns of structural variations and their prevalence in ESCCs are not completely revealed. The clinical significance of these patterns in ESCC is unclear, and driver genes shaped by distinct mutational processes remain largely unknown. To characterize somatic rearrangements and their genomic and clinical implications in ESCCs, we decode the SV signatures based on the size of the SV and rearrangement patterns. We developed a graph-based method to find and classify complex rearrangements. The breakpoints are treated as graph nodes, and two breakpoints of the same SV or two adjacent breakpoints with appropriate orientation are regarded as breakpoint edge and sequence edge, respectively. Taken together with coverage of breakpoints and edge, the underlying rearrangements patterns within ESCCs could be extracted.

In this study, we analyze 528 paired genomes to investigate the potential SV patterns and their clinical implications in ESCC. We define several types of complex rearrangements that are prevalent in ESCCs, of which we highlight a type of fold-back inversion that is associated with poor outcomes. We further explore the mutational process of distinct SV types and uncover their association with genomic metrics. We also identify a hotspot in the super-enhancer of the *PTHLH* gene driven by the TD-c2 signature. Finally, we report the diverse models of TDs and ecDNAs that led to high-level amplification of oncogenes.

## Results

### Data sequencing and characterization of ESCC samples

**Fig. 1:**
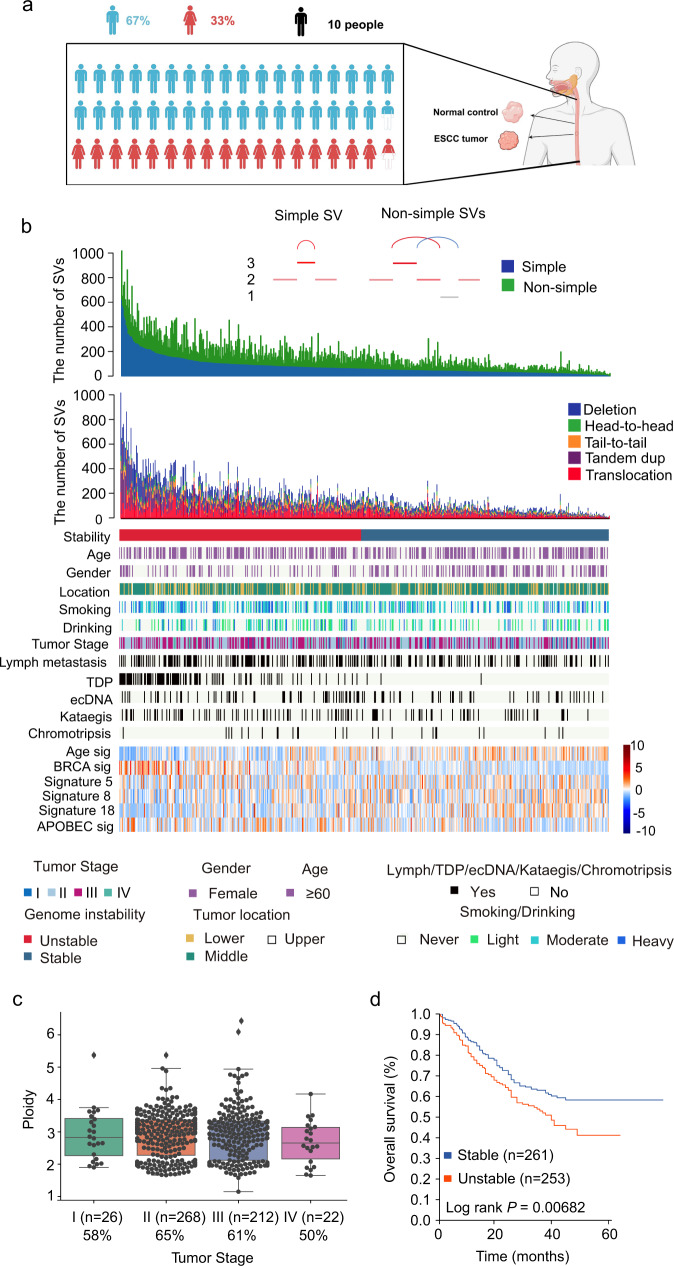
Clinicopathological characteristics and landscape of structure variations in ESCC. **a** The size of cohort for analyses. Created with BioRender.com **b** Top panel shows the number of SVs detected in each genome; middle panel shows key clinical characteristics, and bottom panel shows the contributions of dominant signatures in ESCC. **c** Box plot shows the association between tumor ploidy and tumor stage in ESCC. *n* = 528 biologically independent pairs of samples. On the boxplots the horizontal line indicates the median, the box indicates the first to third quartile and whiskers indicate 1.5× the interquartile range. **d** Kaplan-Meier survival curves show the survival outcomes of stability in ESCC. Statistical analysis is performed with Log-rank test. Source data are provided as a Source Data file.

This study collected 528 tumors and paired normal tissue samples from ESCC individuals, including 395 pairs from Cui et al.^9^. DNAs of these samples were subjected to whole-genome sequencing (WGS) (Fig. 1a). The average coverage for 528 tumor and normal samples were 40× and 25×, respectively (Supplementary Fig. 1a). The median cancer cell content and tumor ploidy of 528 ESCCs were 0.44 and 2.96, respectively (Fig. 1c, Supplementary Data 1). Moreover, 70% of ESCC genomes exhibited GD (genome-doubling) events (Supplementary Data 1), suggesting the high instability of ESCC genomes. Nevertheless, GD events were uniform across different tumor stages, indicating that GD may be a relatively early event during ESCC progression. Interestingly, GD was associated with a favorable prognosis in ESCC (Supplementary Fig. 1b), different from other human tumors^19^. Additionally, patients with advanced-stage ESCCs showed a worse prognosis (Supplementary Fig. 1c).

Svaba^20^ and Delly^21^ were employed to call somatic SVs, and germline and low-quality events were discarded (Supplementary Fig. 2, Method). Consequently, a cohort consisting of 91973 SVs (Supplementary Data 2) was used for advanced analyses. Moreover, to validate the accuracy of our SV cohort, tumor DNAs from three samples (ESCC-064, ESCC-076, and ESCC-260) were re-sequenced, and the results showed that the average accuracy was 87.24%. One tumor had low validation accuracy due to high intratumor heterogeneity (ESCC-064, Supplementary Fig. 3). In addition, the cancer cell content was not associated with SV burden across 528 ESCC genomes. These results suggest that our cohort is suitable for further study.

### SV spectrum and instability of ESCC

An average of 174.2 SVs ranging from 11 to 1018 per genome was obtained (Fig. 1b). The number of each type of SVs varies across individuals. We classify SVs into simple and non-simple SV, and the region between two breakpoints of simple SV did not contain other breakpoints (Fig. 1b, Method). In our data, the number of non-simple SV was varied among 528 ESCC (range: 4–624, Fig. 1b) and accounted for 48% of total SVs. As complex rearrangements involve multiple SVs, disentangling them can be complicated and ambiguous. However, a simple rearrangement involves one SV. Therefore, we used the simple SV burden to estimate the rearrangement burden in each genome. We classified ESCCs into stable and unstable groups according to the simple SV burden median. We found that unstable ESCCs have significantly worse outcomes than stable ones (Fig. 1d). However, the overall SV burden shows insignificance between the two groups (Supplementary Fig. 4a), suggesting using a simple SV burden is a more effective measure than SV burden to reflect patients’ prognosis. We also compared the overall survival of stable or unstable ESCCs of the same tumor stages, and unstable ESCCs with stage IIIB exhibited a poor survival (Supplementary Fig. 4b), indicating that our classification method may help further stratification of advanced ESCCs.

We next examined the association between genome instability and clinical or mutational signatures. Unstable genomes exhibited a high proportion of BRCAness signature, and a low proportion of aging signature (Fig. 1b, Supplementary Fig. 4c), which supports that genome instability could be induced by the deficiency of homologous recombination-mediated double-strand break repair^22^. Besides, compared with the previous report^5^, increased frequency of complex focal events was identified in ESCC (Fig. 1b), such as kataegis (109/528), in which SVs occurred with accompanying local hypermutation. Kataegis was associated with *MDM2* amplification (Supplementary Fig. 4d, *P* = 0.009).

**Fig. 2:**
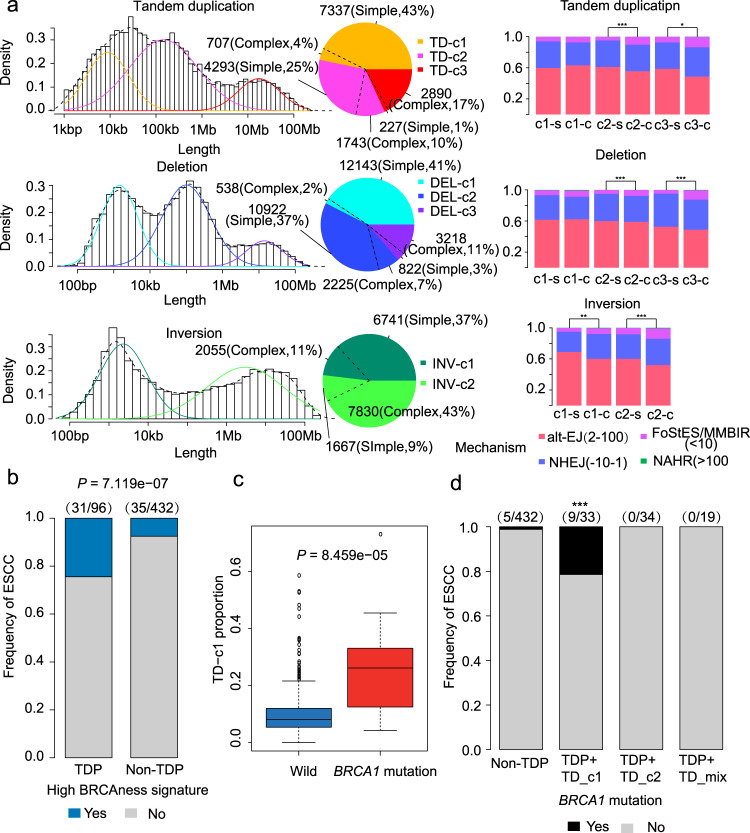
Structural-variant signatures in ESCC. **a** Left panel: Size distribution of tandem duplication, deletion, and inversion. Pie chart shows the frequencies of different SV signatures. Right panel: Bar plot shows the frequencies of different mechanisms of these signatures. The stars represent a significant difference of proportion of FoSTes/MMBIR between two groups. **b** Bar plot shows the enrichment of a high proportion of BRCAness signature in ESCCs with TDP. **c** Box plot shows the high TD-c1 proportion in ESCCs with *BRCA1* mutation. *n* = 528 biologically independent pairs of samples. On the boxplots the horizontal line indicates the median, the box indicates the first to third quartile and whiskers indicate 1.5× the interquartile range. **d** Bar plot shows the enrichment of *BRCA1* mutation in ESCCs with TDP and high TD-c1 proportion. Statistical analysis is performed with a student *t-* test. *P ≤ 0.05, **P ≤ 0.01, ***P ≤ 0.001. Source data are provided as a Source Data file.

Moreover, SVs of distinct mechanisms may have a different size or length of homology sequence within ESCC genomes. We applied GMM (Gaussian Mixture Model) to estimate the peak modes for four types of SVs (deletion-like, TD-like, and inversion: head-to-head/tail-to-tail). We found three modes of TDs at 12 kb(Kilo-base per), 213 kb, and 1170 kb, respectively (Fig. 2a). TD-c1 and TD-c2 were characterized by simple TDs, while TD-c3 tended to be involved in complex rearrangements (Fig. 2a). Interestingly, we found that 96 (17%) ESCC genomes had overrepresented TDs (Fig. 1b), and they could be classified as Tandem Duplication Phenotype (TDP)^23^. The proportion of TD-c1 and TD-c2 in TDP ESCCs was significantly higher than that in non-TDP ESCCs (Supplementary Fig. 4e), while TD-c3 was lower in TDP ESCCs than non-TDP ones. In addition, 31/96 TDP ESCCs harbored BRCAness signature. The contributions of BRCAness signature were markedly higher than that in non-TDP cases (35/432, Fig. 2b). We, therefore, investigated the association between TD signature and *BRCA1/2* variants. TD-c1 proportions significantly elevated in *BRCA1*-mutated ESCCs and decreased in *BRCA2*-mutated ESCCs (Fig. 2c and Supplementary Fig. 4f). *BRCA1* variants were highly enriched in TDPs with a high TD-c1 proportion (Fig. 2d). These results suggested that TDP with a high TD-c1 proportion may be a feature of *BRCA1* variants in ESCC, consistent with previous studies^24,25^. As we have reported the role of *BRCA1/2* in platinum therapy of ESCC^8^, further studies need to validate whether ESCCs with TDP and high TD-c1 can benefit from platinum-based chemotherapy.

Furthermore, there were three deletions modes located at 1.7 and 105 kb and 9.7 Mb (Megabases), respectively (Fig. 2a). DEL-c1 and DEL-c2 were predominant with simple deletions, and DEL-c3 was associated with complex rearrangements. In addition, two inversion peaks were identified at peaks 3 kb and 4.1 Mb (Fig. 2a). By analyzing the microhomology, we identified that alt-EJ (Alternative end-joining) and NHEJ (Non-homologous end joining) were the primary repair mechanisms for both three types of simple SV (Fig. 2a). In contrast, FoSTes/MMBIR (Fork stalling and template switching/ Microhomology mediated break-induced replication) mechanism was more frequent in complex events (Fig. 2a).

### TD-driven hotspots in ESCCs

**Fig. 3:**
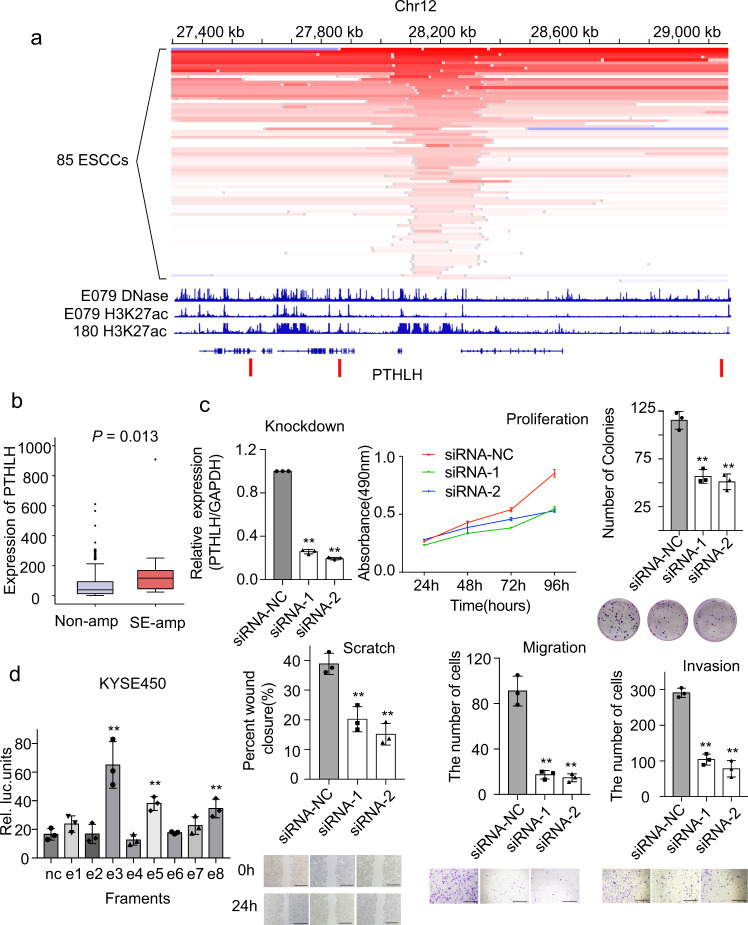
Driver gene *PTHLH*. **a** Focal amplifications in 85 ESCCs around gene *PTHLH* were shown. DNase (first line) and H3K27ac (second line) data from one sample E079 in RoadMap are shown. KYSE-180 H3K27ac (third line) and TADs (last line) boundary of GM12878 are also shown below. **b** Box plot displays the *PTHLH* expression in ESCCs grouped by super-enhancer amplification. *n* = 111 biologically independent samples. On the boxplots the horizontal line indicates the median, the box indicates the first to third quartile and whiskers indicate 1.5× the interquartile range. **c** Cell function experiments of *PTHLH* in ESCC cell line KYSE180. Upper left: Bar plot displays the knock-down efficiency of *PTHLH*. Upper right: Line plot displays the proliferation of cells after *PTHLH* knock-down by MTT assay and bar plot displays the number of colonies in cells. Lower left: Cell-migration monitored by wound healing assay and bar plot displays migration result. Lower right: The Transwell cell migration and invasion assay after *PTHLH* knock-down. Scale bars, 500 μm. All bar-plot are presented as the mean ± standard deviation. Three independent experiments were performed; each experiment was performed in triplicate. Statistical analysis is performed with one-way ANOVA. **d** The bar plot shows results of dual luciferase reporter assay. Luciferase-reporter assays measuring the activity of e1 to e8 in KYSE450 cells. The pGL4.10-E4TATA plasmid without the enhancer region was used as a control. Bar-plot are presented as the mean ± standard deviation. Three independent experiments were performed; each experiment was performed in triplicate. Statistical analysis is performed with one-way ANOVA. *P ≤ 0.05, **P ≤ 0.01. Source data are provided as a Source Data file.

To systematically investigate TD hotspots in ESCC, we applied methods proposed by Glodzik^23^ to distinct TD signatures. As a result, we identified 9 TD-c2 hotspots and 4 TD-c1 hotspots (Supplementary Table 1). In addition to well-established hotspots such as *KLF5* and *MYC*, the data also reveal a hotspot consisting of *PTHLH* (Fig. 3a). To verify the accuracy of TDs consisting of *PTHLH*, 11 TDs were subjected to Sanger-PCR validation. Experimental data confirmed that these TDs were true somatic events (Supplementary Fig. 5). Importantly, these TDs did not disrupt the TAD (topology-associated domain) boundaries (Fig. 3a). In our data, 85 out of 528 (15%) ESCCs harbored amplification of *PTHLH* (Fig. 3a). Specifically, amplifications of *PTHLH* were predominated with low copy number gain (Fig. 3a). Complex rearrangements with template switch events lead to low-copy number gain of *PTHLH* in four ESCCs (Supplementary Fig. 6). We also noticed that most amplifications were involved in the upstream of *PTHLH* and had an overlapped region. The chromatin data of DNase-seq and HK27ac from normal esophagus tissue and KYSE-180^26^ were inspected and implicated an active enhancer in the overlapped region (Fig. 3a). The super-enhancer of *PTHLH* is located in the overlapped region^26^. Hence, *PHTLH* is probably a cancer-associated gene driven by super-enhancer amplification in ESCC.

*PTHLH* is a protein-coding gene associated with cellular and organ growth, development, and migration^27^. It has been previously reported to promote cancer cell proliferation and invasion in head and neck cancer^28^. Its biological function in ESCC remains unknown. In two different ESCCs cohorts, mRNA (*n* = 133) and protein (*n* = 134) expression of *PTHLH* in tumors were significantly higher than that in normal tissue (*P* = 2.54E−14 & 0.00029, respectively, Student’s *t*-test, Supplementary Fig. 7a). In our ESCCs RNA-seq cohort (*n* = 133), tumors with genebody or super-enhancer amplification of *PTHLH* displayed higher mRNA expression than that in tumors without amplification (*P* = 0.031, Student’s *t*-test, Supplementary Fig. 7b). Further exploration showed that the *PTHLH* expression of tumors with super-enhancer amplification was still significantly higher than tumors without amplification (*P* = 0.013, Student’s *t*-test, Fig. 3b). Knockdown of *PTHLH* significantly inhibited cell proliferation, colony formation, cell migration and invasion (Fig. 3c, Supplementary Fig. 8). These data suggested that *PTHLH* was probably a cancer gene in ESCC. Further, to investigate whether super-enhancer amplifications drive the expression of *PTHLH*, we inserted one or double fragments (high-intensity signal in H3K27a) of the super-enhancer region of *PTHLH* into the vector of a dual-luciferase reporter gene. The result showed that the super-enhancer region could significantly promote the expression of the reporter gene, and doubled enhancers showed higher transcriptional activity than that of one copy enhancer. (Fig. 3d, Supplementary Fig. 7c). To investigate the functional role of the e3 and e5 enhancer region in *PTHLH* expression, we deleted the e3 and e5 enhancers using the CRISPR/Cas9 system, respectively. Deletion of e3 and e5 was detected by PCR (Supplementary Fig. 7d). The data showed that deletion of the e3 and e5 enhancer region resulted in decreased *PTHLH* expression (Supplementary Fig. 7e). These findings suggest that *PTHLH* is a putative driver primarily driven by super-enhancer amplifications in ESCCs.

### Complex rearrangements prevalent in ESCCs

**Fig. 4:**
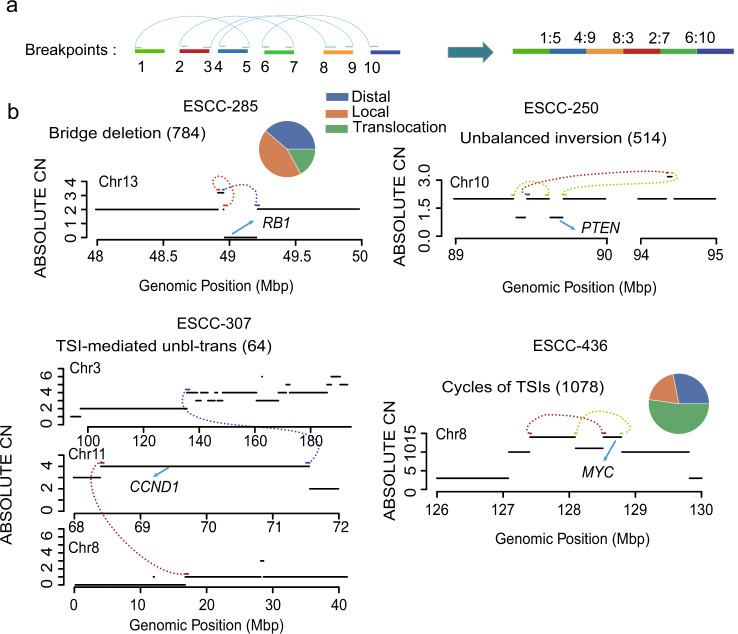
Complex rearrangements that prevalent in ESCCs. **a** An example illustrates the method of how to construct potential complex rearrangement. **b** A diagram of three types of complex rearrangement. The number for each complex rearrangement is marked. The pie chart shows the type of TSI near to start or end breakpoint of complex rearrangement. The black solid lines represent the copy number states of the target region, whereas structural variations are shown as colored dotted lines (brown: breakpoint strand “--”; green: breakpoint strand “++”; purple: translocation) linking two segments. Source data are provided as a Source Data file.

Complex rearrangements involve two or more SVs and lead to newly genomic segment. It hence could be thought of as a combination of a simple SV and cycles of TSIs (Templated sequence insertions). Based on this concept, we developed a graph-based method to infer and classify complex rearrangements based on SVs’ position, orientation, and normalized coverage within each ESCC genome (Fig. 4a, Method). In brief, all breakpoints within each genome were nodes. Any two breakpoints from the same SV or two adjacent breakpoints with appropriate rearrangement orientation in the chromosome were connected by an edge. A new genomic segment consisting of four or more breakpoints was probably arising from complex rearrangements, and these segments were further refined to identify complex rearrangements.

Of these segments, we identify 784 bridge deletions (also called the bridge of templated sequence insertions (TSIs)), 514 unbalanced inversions, and 1079 cycles of TSIs (also called circular TD), and 64 TSI-mediated unbalanced translocations, respectively (Fig. 4b, Supplementary Data 3). To verify the accuracy of the method, we applied it in two paired ESCC genomes with Nanopore and NGS data. 4 out of 5 complex rearrangements could find the supporting Reads from Nanopore (Supplementary Fig. 9), suggesting high accuracy of the method. The number of these complex events varied across ESCC individuals and tended to enrich in unstable genomes (Supplementary Fig. 10a). Size distribution of TSIs demonstrated two peaks that were lower or higher than 10 kb, respectively, within both bridge deletions and cycles of TDs (Supplementary Fig. 10b). The large peak was characterized by similar size distribution to that of coding genes, suggesting that they might drive oncogene expression by increasing extra copy numbers. It was noted that 81.7% of TSIs from bridge deletions have a size less than 10 kb (Supplementary Fig. 10b), significantly higher than that from cycles of TSIs (40.4%). Moreover, compared to TSIs of bridge deletions, cycles of TSIs were more likely from different chromosomes, while TSIs within bridge deletions events were primarily from local genomic regions (Fig. 4b).

We further examined complex rearrangements involving ten or more SVs. A total of 448 potential complex events involved in 231 ESCCs were identified. Seventy-four complex events were characterized by a series of inter-chromosomal SVs (Supplementary Fig. 11). One hundred fourteen events displayed a series of oscillations between 2 or 3 copy number states, suggesting that these chains result from chromothripsis. Chromothripsis is formed through chromosomal shattering followed by random re-joining or template-switching events such as FoSTes/MMBIR^18,29^. It was a challenge to distinguish between them in our data since most ESCC genomes demonstrated high ploidy or neutral LOH(Loss of heterozygosity) within affected chromosomal arms. Thus, only a few of them displayed pronounced LOH. In our data, there was a total of 93 chromothripsis events identified in 77 ESCCs (Supplementary Data 4). Although all these events displayed oscillating copy numbers, segment size and genomic distribution were variable and could be summarized as three patterns (Supplementary Fig. 11). The first kind of chromothripsis displayed dozens of oscillated copy numbers of large size (usually several Mb) with uniform distribution across a chromosomal arm, which was consistent with canonical chromothripsis (Supplementary Fig. 11b). The second displayed the small size of segments (usually around 100 kb) preferentially occurring in one or multiple localized regions (Supplementary Fig. 11c). The third one displayed a similar size to segment compared to the second one but fewer SVs and short spanning genomic region compared to the first pattern (Supplementary Fig. 11d). These three patterns accounted for 46 (8.7%), 19 (3.6%), and 28 (5.3%) ESCCs in our data, respectively.

### A type of fold-back inversion associated with poor outcome

**Fig. 5:**
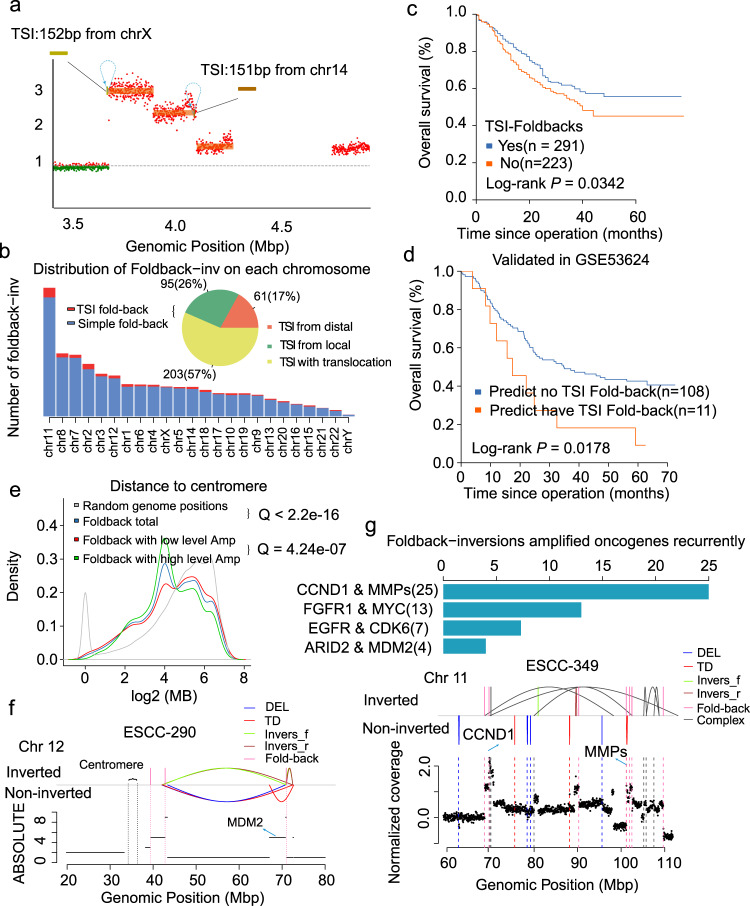
Landscape of TSI fold-back inversions in ESCC. **a** An example of TSI fold-back inversion in ESCC. **b** Bar plot shows distribution of fold-back inversion on each chromosome. Pie chart shows the frequencies of three types of TSI fold-back inversion. **c** Kaplan-Meier survival curves shows the survival outcomes of TSI fold-back inversion in ESCC. Statistical analysis is performed with Log-rank test. **d** Kaplan-Meier survival curves shows complex fold-back have a similar worse prognosis in validation data. Statistical analysis is performed with Log-rank test. **e** Density plot shows association between fold-back inversion and centromere. **f** A specific example shows fold-back inversion be close to centromere not only in physical distance but also in actual distance. **g** Bar plot shows spectrums of fold-back inversions amplified genes recurrently. Bottom panel shows an example that *CCND1* and *MMP*s on chromosome 11 were amplified by fold-back inversions synchronously. Source data are provided as a Source Data file.

We noticed a small proportion of genomic segments with two breakpoints of the same orientation, less than 100 kb. These two breakpoints were connected by cycles of templated insertions that usually displayed high-level copies (Fig. 5a). Especially, telomere deletion was generally present with these patterns, and they also displayed the stepped shape in copy number profiles (Fig. 5a). These features were similar to fold-back inversions except for templated insertions; hence, we defined it as TSI fold-back inversion. In our cohort, we totally identified 359 TSI fold-back inversions, which could be classified into three categories based on the location of the inserted templates. The inserted segments from the distal (17%, >1 Mb) and local (26%, <1 Mb) region of the same chromosome with the fold-back inversions were shown in Fig. 5b and Supplementary Fig. 12a, b. More than half (57%) of TSI fold-back inversions were companied with unbalanced translocations, indicating genomic segments from other chromosomes joined through templated insertions. In Supplementary Fig. 12c, the segment from chromosome 12 is inserted into BFB cycles on chromosome 11. Moreover, TSI was generally smaller than 1 kb (Supplementary Fig. 12d). These TSIs were more likely to be derived from highly expressed genomic regions and closer to triplex mirror repeats (Supplementary Fig. 12e). Triplex forming mirror repeats were reported to induce genome instability in the previous study^30^, and the relationship between these repeats and TSI fold-back inversions needs further investigation. Furthermore, 42% ESCCs having TSI fold-back events displayed a worse prognosis (Fig. 5c). We constructed a gene expression signature for TSI fold-back inversions to validate the prognostic ability of TSI fold-back inversions using 133 tumors with available RNA-seq data. A random forest classifier could detect ESCC with complex fold-back inversions by using 598 DEGs. Ten repeats of 10-fold cross-validation in our data set led to the optimal selection of 185 DEGs (Supplementary Data 5) that demonstrated the lowest classification error and an area under the receiver operating curve (AUC) of 83.07% (Supplementary Fig. 13). The classifier was then applied in another independent 119-ESCCs cohort^31^ (GSE53625) and showed that the predicted ESCCs with complex fold-back had a similar worse prognosis (Fig. 5d). Together, our results suggest that complex fold-back inversion may be a potential prognostic biomarker for ESCCs.

We next examined simple fold-back inversions and derived patterns across ESCC genomes. By applying the method described in Cambell et al.^32^, we identified a total of 5429 simple fold-back inversions (Supplementary Data 6), most frequent in chromosomes 11, 8, and 7 (Fig. 5b). Correlating fold-back inversions with genomic properties, we found that fold-back inversions occurred preferentially around the centromere and enriched in high-level amplification (Fig. 5e). Primarily, in the case of ESCC-290 with two BFB cycles on chromosome 12, one occurred near the centromere, and another occurred on the distal end of the chromosome arm but displayed close spatial proximity to the centromere, which was connected by complex rearrangements, reinforcing the hypothesis that fold-back inversion tends to occur near the centromere (Fig. 5f). Moreover, fold-back inversions tended to occur in male patients and upper regions of the esophagus. They were associated with lymphatic metastases, TNM stage, and mutational signatures such as BRCAness and APOBEC-mediated signature (Supplementary Fig. 14a, b). ESCCs with more fold-back inversions (>5) were moderately associated with poor prognosis (Supplementary Fig. 14c). Fold-back inversions were also positively correlated with simple deletions (Supplementary Fig. 14d), indicating that fold-back inversions were likely to occur in cases with a high number of double-stranded breakage.

In the previous study^10^, we proposed that alt-EJ might be the primary underlying mechanism for the formation of BFB cycles. This trend was consistent in the current data set, except for the proportionally larger of FoSTes/MMBIR (insertion >10 bp), which was probably owing to TSI fold-back (Supplementary Fig. 15). Following the fold-back capping of broken ends and fusion of sister chromatids, BFB cycles could lead to high-level amplification of genomic regions, especially for well-known oncogenes^33^. Apart from previously identified *CCND1*, *EGFR*, *ERBB2*, *MMP*s, and *MYC*, we also observed many oncogenes such as *FGFR1, MDM2*, and *FOXA1* amplified by BFB cycles (Supplementary Fig. 16). These BFB cycles of oncogene were also associated with clinical factors, prognosis, and mutational signatures (Supplementary Fig. 14a and Supplementary Fig. 17). Moreover, we found that *CCND1*, *FGFR1*, *MMP*s, and *MDM2* BFBs were more likely to have a high-level amplification (CN **>** 10), whereas *TRO*, *ARID2*, *TEC*, and *ERBB2* BFBs resided in the low-level amplification (CN **≤** 10) region. Interestingly, we observed that oncogenes from the same chromosome might be amplified by fold-back inversions synchronously, such as *CCND1* with *MMPs* on chromosome 11 (*n* = 25) and *EGFR* with *CDK6* on chromosome 7 (*n* = 7) (Fig. 5g and Supplementary Fig. 18). This pattern of recurrent amplification of two oncogenes was connected by complex rearrangements through one genomic chain, suggesting a special fusion pattern of amplified oncogenes derived from fold-back inversion and complex rearrangements in the evolutionary trajectory of ESCC. Fold-back inversions around *MMPs* were placed within close spatial proximity to the centromere through this pattern. Nevertheless, the exact time order of fold-backs and complex rearrangements in the formation of the particular fusion was unknown. We did not find an association between this particular pattern and clinical factors in our cohort.

### Diverse patterns of somatic rearrangements that affect driver genes

**Fig. 6:**
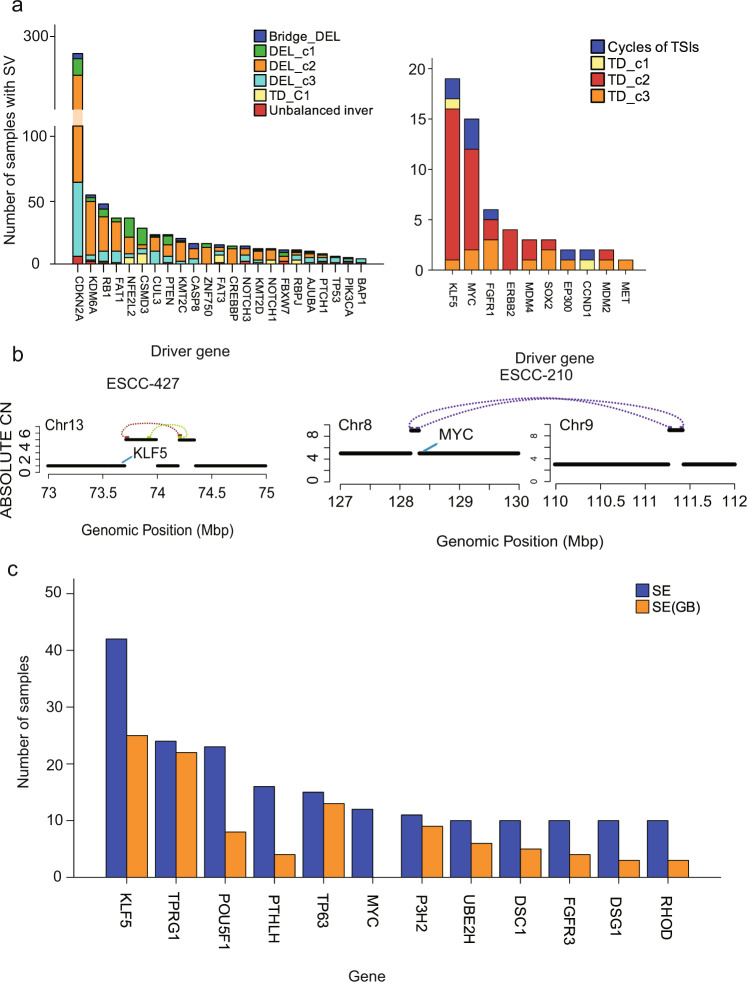
Driver genes by SV in ESCC. **a** Stacked Bar plot shows the SV types of driver genes. Left: Tumor suppressors in ESCC. Right: Oncogenes in ESCC. *n* = 528 biologically independent pairs of samples. **b** Examples: Amplifications of *KLF5* super-enhancer and *MYC* super-enhancer owing to Cycles of TSIs. The black solid lines represent the copy number states of the target region, whereas structural variations are shown as colored dotted lines (brown: breakpoint strand “--”; green: breakpoint strand “++”; purple: translocation) linking two segments. **c** The bar plot show the gene enhancer regions are most affected by simple TD. Blue indicates that both enhancer and gene regions are affected, and orange indicates that only enhancer regions are affected. *n* = 528 biologically independent pairs of samples. Source data are provided as a Source Data file.

By increasing or deleting gene copy numbers, SVs affect the expression levels of certain genes, thus promoting tumor initialization or progression. We first examined the ESCC-associated genes affected by simple TD and DEL. *KLF5* (15/528), *MYC* (10/528), and *ERBB2* (4/528) were frequently amplified by TD-c2 (Fig. 6a). In addition, TD-c1 tended to truncate genes such as *NOTCH1* and *FAT3* while they amplified *CCND1*, indicating the dual rule of TD-c1. Compared to Del-c1 and c3, the majority of cancer genes were deleted by DEL-c2, such as *CDKN2A* (207/528), *FHIT* (133/528), *KDM6A* (42/528), *RB1* (27/528), *FAT1* (23/528), *NFE2L2* (13/528), *ERBB4* (20/528), *CUL3* (11/528), *PTEN* (9/528), *ZNF750* (13/528) and *NOTCH1* (8/528) (Fig. 6a).

Regarding complex rearrangements, we found that *CDKN2A* (6/528), *FHIT* (6/528), *KMD6A* (2/528), *PTEN* (1/528), *RB1* (1/528), *TP53* (1/528), and *FAT1* (1/528) were affected by unbalanced inversions (Fig. 6a), and *CDKN2A* (4/528), *RB1* (4/528), *FHIT* (4/528), *KDM6A* (2/528), *NOTCH3* (2/528), *FBXW7* (2/528), *CUL3* (1/528), *PTEN* (1/528) and *AJUBA* (1/528) were involved in bridge deletion (Fig. 6a). Interestingly, 6/10 *CDKN2A* deletions arising from complex events were precisely owing to unbalanced inversions (Supplementary Fig. 19). Among four bridge deletions of *CDKN2A*, three of which displayed two cycles of TSIs. For ESCC oncogenes, *MYC* (3/528), *KLF5* (2/528), *FGFR1* (1/528), *EP300* (1/528), and *CCND1* (1/528) were amplified by cycles of TSIs (Fig. 6a). It was worth noting that, of 6 complex events involving *MDM2*, four belonged to unbalanced translocations with TSIs (Supplementary Fig. 20), indicating that *MDM2* was more likely to be amplified by unbalanced translocations compared to other complex events. Specially, we found a TSI consisting of *CD274* in ESCC-437 (Supplementary Fig. 21a). We reported the high-level amplification of *CD274* and associated expression in ESCC previously^34^. In this cohort, we found 10/528 ESCCs harbored *CD274* amplification (Supplementary Fig. 21b), which further confirmed our previous conclusion, and this type of patient might be more sensitive to immune checkpoint blockade. Chromothripsis affected multiple driver genes, including oncogenes such as *CCND1* (3), *ERBB2* (2), *PIK3CA* (4), *SOX2* (5), *MYC* (4), *CASP8* (3), *MDM4* (1), *CD274* (1), and tumor suppressor genes such as *RB1* (1), *CUL3* (1) and *ZNF750* (1) (Supplementary Data 4).

In addition to the gene body regions, TDs also affected the copy number amplification of gene regulatory regions, such as enhancers. The enhancer, instead of the gene-body region of genes, was frequently amplified by simple TD, such as *KLF5* and *TP63*, (Fig. 6c). We also noticed that regulatory elements of driver genes were frequently amplified by cycles of TSIs. Specifically, enhancers of genes *MYC* and *KLF5* were amplified by cycles of TSIs (Fig. 6a, b, and Supplementary Fig. 22a, b). These data imply that TD events could affect the gene body and regulatory elements to regulate cancer gene expression.

### Genomic properties and clinical significance of diverse rearrangements

**Fig. 7:**
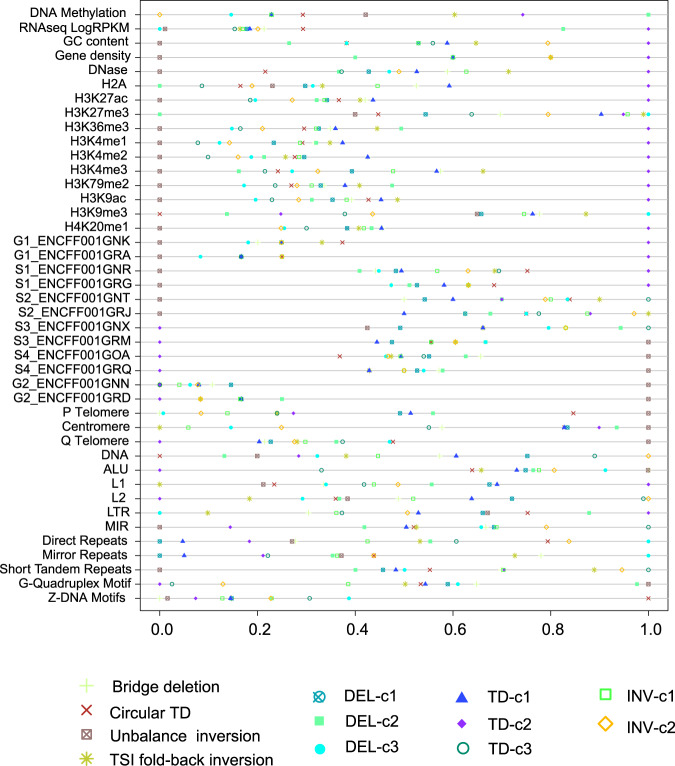
Comparison of the median values of genomic metric across different SV patterns. Chromosomal regions are divided into non-overlapping 1k window and the values of each metric are estimated based on 1k window. The median values for different SV patterns are normalized to [0, 1]. Source data are provided as a Source Data file.

To assess how these patterns associate with genomic metrics, we compared a series of features such as DNA-replication time, chromatin accessibility, and the distance of repeats nearby among distinct SV patterns. The median values for each feature across distinct SVs were scaled between 0 and 1, as shown in Fig. 7. Firstly, we found that TD-c2, unbalanced deletions exhibit pronounced separation of genomic metrics from other SVs. Specifically, the TD-c2 signature demonstrated enrichment in the region of early DNA-replication time, chromatin accessibility, and high-expression region (*P* **<** 1e−06). In contrast, deletion events such as unbalanced inversions and bridge deletion were likely to occur in the region of late DNA replication time (*P* **<** 1e−06). Secondly, TD-c3 was more likely to occur in the region of the S stage during DNA replication. TD-c2 and -c3 tended to occur near L1 (LINE-1), L2 (LINE-2), LTR (Long-Terminal Repeats), and MIR (Mammalian-wide interspersed repeat). Thirdly, fold-back inversion is more likely to occur near the centromere, which agrees with those mentioned above. Finally, DEL-c2 occurred in a high-methylated region compared to other events.

We also observed an association between SV signatures and mutated genes, of which *FAT2* and *JAG1* mutations were more likely to occur in ESCCs with a high proportion of TD-c2 and DEL-c2, respectively. In contrast, *FBWX7, KDM5A, NOTCH1*, and *PIK3CA* mutations were repressed in ESCCs with a high proportion of TD-c3, DEL-c3, INV-c1, and INV-c2, respectively (Supplementary Fig. 23). These findings suggest that distinct SV processes may have variable genomic properties and impacts on genetic mutations.

### Underlying mechanisms for high-level amplifications

**Fig. 8:**
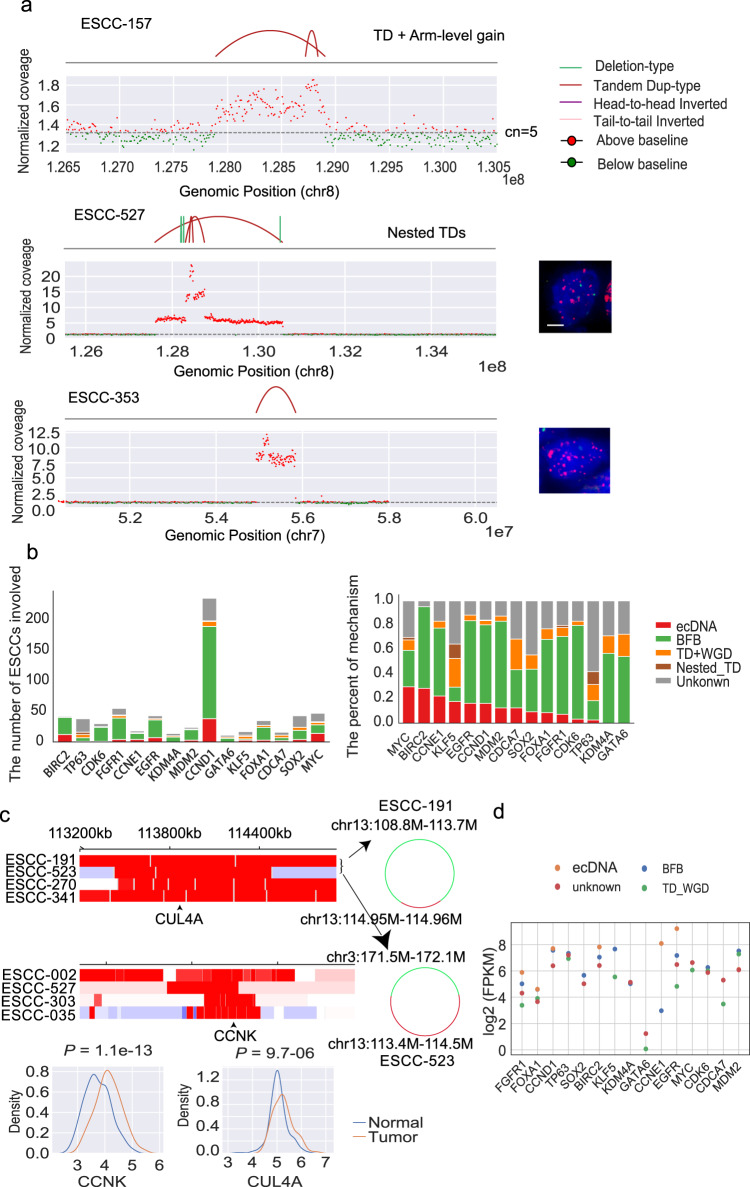
Underling mechanism for high-level focal amplifications. **a** The structural variations and normalized coverage for TD-dominated amplicons. Right: Representative immunofluorescence images show signals produced from FISH analyses using probes specific to chromosome (red) and target gene (green) in ESCC sample. Scale bars, 5 μm. **b** ESCC cancer genes affected by different mechanism. Left panel shows the number of ESCCs involved in amplified genes; right panel displays the proportion of mechanism for amplification. **c** Genes affected by ecDNA. Heatmap show normalized coverage based on IGV. Some ecDNAs are constructed manual and shows in right panel. The expression of two potential cancer-associated gene (*CCNK* & *CUL4A*) in 133 paired RNA-seq data shown in bottom. Statistical analysis is performed with student *t*-test. **d** The average expression of genes across different samples affected by each mechanism. Source data are provided as a Source Data file.

High-level amplifications usually harbor oncogenes essential for tumor progression. To investigate underlying rearrangements that resulted in high-focal amplifications, we identified 2559 amplified segments with ≥five copies than baseline. 565 segments were lack of structural variations, which were probably underestimated by SV calling software. The remaining segments totally yield 1994 amplicons, consisting of 1641 single, 193 multi-intrachromosomal, and 160 multi-interchromosomal amplicons (Supplementary Data 7). 50.6% (1009/1994) amplicons harbored fold-back inversions indicating the presence of BFB events, which suggested that BFB was a major mechanism for oncogene amplification in ESCC. Interestingly, we found that 18.0% (358/1994) was predominated by TD events. Although TD generally leads to one extra copy number, it will lead to high-level focal amplifications when aggregated with other duplication events such as genome doubling, and arm-level gain. This aggregation seems prevalent in ESCCs genomes due to a high frequency of genome doubling and neutral LOH events. One example of such a case with high-level *MYC* amplification was shown in Fig. 8a, and TD events are enlarged by arm-level gain.

Of these 358 amplicons driven by TDs, 11 showed extremely high copy numbers suggesting the presence of ecDNA events (Fig. 8a and Supplementary Fig. 24). EcDNA demonstrates a circular genomic structure with high-level amplification. Therefore, we rely on CN (copy number) and choose amplified regions with CN **>** = 5 mentioned above to make our inferences. This criterion is frequently used in many previous studies^15,29,35^, such as AmpliconArchitect^35^, to infer ecDNA. In addition, we employed AmpliconArchitect for each amplicon, which requires a circular structure within the amplicon (Method). 99 (17.5%) ESCCs were likely to harbor ecDNA events. Of which ecDNA involved in 80% ESCCs showed simple cycles using AmpliconArchetect^15^, indicating the accuracy of these ecDNA events. Moreover, we randomly choose 49 ecDNA events for validation using the FISH experiment. 75.5% ecDNA events showed the strong, scattered intensity of probe signals (Supplementary Fig. 25).

Consistent with previous studies, high-level amplifications frequently affected driver genes such as *CCND1*, *EGFR*, *CDK6*, and *ERBB2* (Supplementary Fig. 26a). *CCND1* was frequently involved in ecDNA, accounting for 44% (33/75), followed by *MYC* (13%) and *MMPs* (8%). 5% *KLF5* and 8% *TP63* amplicons were from nested TDs (Fig. 8b). Some ecDNAs consisted of multiple cancer genes within one event. For instance, ecDNA in a sample consisted of cancer gene *MYC*, *CCND1*, and *MMPs*. Interestingly, ecDNA-driven genes might have preferential partners, as ecDNA of *CCND1* is more likely to connect with *MMPs*. In addition to well-established cancer genes involved in ESCC, there were two ecDNA-amplified regions located at chromosome chr13:113-114 Mb consisting of gene *CUL4A* and chr14:100 Mb consisting of gene *CCNK* (Fig. 8c). Of 133 samples with RNA-seq data available, *CUL4A* and *CCNK* expression of the tumor was significantly higher than that in normal tissue (Fig. 8c).

In contrast to recent studies^15^, ecDNA events were not associated with patient outcome in ESCCs (Supplementary Fig. 26b). The mutation rate of the genomic region involved in ecDNA was significantly higher than that of the whole genome (Supplementary Fig. 26c). 85% of somatic mutations involved in ecDNA demonstrated low variant allele frequency (VAF **<** 0.1), indicating that most ecDNA mutations arise after ecDNA formation (Supplementary Fig. 26d). Taken together with the observation that ecDNA event was uniformly distributed across ESCCs with different tumor stages (Supplementary Fig. 26e), we hence anticipated that ecDNAs occur early during ESCC evolution. To explore the expression level of genes involved in amplicons, we employed 133 ESCCs with RNA-seq data to compare the expression of genes subjected to different mechanisms. More than 30% of genes involved in BFB demonstrate elevated expression in tumor tissues compared to that in normal tissue (Fold-change ≥1, Supplementary Fig. 26f). The expression of BFB-derived genes is comparable to that of ecDNA genes (Supplementary Fig. 26g). However, when we only inspected the expression level of driver genes, genes involved in ecDNA exhibited higher expression than that in BFB (Fig. 8d). These results suggest ecDNAs might have stronger selective advantages to driver genes than BFB.

## Discussion

In this study, we present diverse patterns of simple and complex rearrangements. These patterns harbor distinct genomic and clinical properties, suggesting specific biological mechanisms drive them. In our data, *BRCA1* variants are associated with short TDs (TD-c1) and TDP. As therapy on BRCA variants is deeply explored in breast and ovarian cancer, this result replicates the importance of *BRCA1/2* detection for ESCC treatment. Moreover, some of the patterns are significant driver sources, such as TD-c2, DEL-c2, fold-back inversion, and ecDNA, et al. TD-c2 has a remarkable distinction from others and is more likely to occur in the early-replicating and chromatin-accessibility region. Although the causal mechanisms are unclear, future work might focus on understanding these processes and designing drugs based on them.

BFB events frequently occur in ESCC and lead to high-level amplification of oncogenes. We noticed that most oncogenes amplified by BFB events are likely from the genomic region near the centromere, such as genes *CCND1*, *EGFR*, *FGFR1*, and *ERBB2*. Although some fold-back inversions are far from the centromere, we observed that a proportional fold-back inversion is spatially close to the centromere, which distal SV, such as deletion cause. In addition, we also notice that fold-back inversions frequently occurred in centromere such as chromosomes 2 and 4, but no drivers are reported in these regions. These data probably indicate that the genomic site of fold-back inversion is not random or only due to driver genes’ selective advantage. More other factors are needed to explain its preference for the centromere region.

We develop tools to understand complex rearrangements across 528 genomes. Although there are a relatively small number of oncogenes and suppressors affected by complex events, we notice that some genes are more likely to be influenced by specific patterns. For instance, *CDKN2A* deletions are more likely to arise from unbalanced inversion than bridge deletion; 4 out of 6 *MDM2* amplifications result from unbalanced translocation. Additionally, owing to the limitation of next-generation sequencing, the sensitivity of the somatic SV cohort is still incomplete. Thus, it is challenging to identify the intact patterns and their prevalence in ESCC genomes, especially in focally amplified regions with dozens of breakpoints. Consequently, we probably underestimate the prevalence of complex rearrangements defined in the present study. In addition, we might also underestimate sub-clonal rearrangements owing to low supporting reads, such as the low-copy number of ecDNA.

Finally, in our data, both simple TDs and TSI-related rearrangements demonstrate low-copy number gain in the regulatory region. In addition to well-established genes *KLF5* and *MYC*, we also nominate a gene, *PTHLH*, which was primarily driven by super-enhancer amplifications. Previous ESCC studies focus on the coding region, and the regulatory part seems to be a new area for ESCC study.

## Methods

### Sample selection and clinicopathological features of ESCC

A total of 528 patients were from two clinical centers in Shanxi and Xinjiang provinces. All subjects obtained informed consent, and the study was approved by the Institutional Review Boards of Shanxi Medical University and Shanxi Cancer Hospital (Shanxi, China). Each tumor specimen had a paired normal counterpart. All tumors were classified based on WHO criteria. At least three independent pathologists reviewed each sample’s H & E stained sections to confirm that the tumor specimens were histologically consistent with ESCC and that the adjacent tissue specimens did not contain tumor cells. The clinical stage of cancer was determined by the TNM staging system (Eighth Edition) of the international alliance against cancer (UICC)/American Joint Committee on Cancer (AJCC). The clinical backgrounds for this cohort are shown in Supplementary Data 1. We have obtained informed written consent from all the patients in our study.

### DNA extraction and whole-genome sequencing

The total DNA was extracted from FFPE samples. According to the manufacturer’s instructions, high-quality total DNA was extracted by Maxwell 16 tissue DNA Purification Kit (Promega). Approximately 300 ng high-quality DNA samples (od260/280 = 1.8 ~ 2.0) were cut to ~350 BP with Covaris S220 Sonicator (Covaris). Sample Purification Beads (Illumina) was used to purify fragmented DNA. Adapter-ligated libraries were prepared with the TruSeq Nano DNA Sample Prep Kits (Illumina) and sequencing was performed by an Illumina HiSeq system for 2 × 150 paired-end sequencing.

### RNA-sequencing

Total RNA was extracted from frozen samples using Trizol reagent (Life Technologies, Carlsbad, CA, USA) and DNA was digested with DNase I according to the manufacturer’s instructions. The quantity and quality of RNA were evaluated by Nanodrop Spectrophotometer (Thermo Scientific, USA). RNA integrity was measured by 1% gel electrophoresis. mRNA was enriched with oligonucleotides (DT) and cleaved into fragments to prepare the cDNA library. The quality of cDNA library was checked by Agilent 2100 biological analyzer and ABI Step One Plus Real-Time PCR System, and then sequenced on Illumina Hiseq X Ten.

### Long reads sequencing

We used ONT(Oxford Nanopore Technologies) platform for long reads sequencing. ONT sequencing is a new generation of nanopore-based single-molecule real-time electrical signal sequencing technology^36^. Total DNA was extracted from fresh samples. ONT library was constructed using the PromethION platform with the highest throughput. After the sample was qualified, the DNA could be fragmented by the Megaruptor, and the long DNA fragment was enriched (15 kb above) and purified. Next, end repair and dA-tailing were performed with the purified DNA. After purification, the ONT standard sequencing adapter, motor protein, and tether protein were ligated; the prepared DNA library was subjected to a library quality check and sequenced on the machine.

### Long reads sequence filtering and quality control

The resulting data was called Raw Data or Raw Reads, which was stored in FASTQ (referred to as fq) format. NanoPack^37^ was used for subsequent data processing, a set of tools developed for visualization and processing of long-read sequencing data from Oxford Nanopore Technologies and Pacific Biosciences, including a series of sub-tools. We used nanoplot to perform data quality control, and we used the nanopack software to filter the pass reads with Q-score greater than 7 and removed the reads with lengths less than 500 bp. The final data was clean reads.

### Long reads sequences alignment and SVs validation

Minimap2^38^ was used to map DNA sequences against the human reference. In addition, we also applied Minmap2 in aligning the sequence caused by SV obtained from SvABA to long read sequences, so as to verify the accuracy of SVs of the short sequencing. The validation principles are as follows:

1. For simple SVs, we aligned the rearranged sequence to ONT data. If more than 80% of the continuous bases are aligned successfully in tumor samples but not in normal control samples, these SVs would be defined as positive SVs.
2. In addition, the condition for successful verification for complex rearrangments is that the rearranged sequence, including all SV junction sites and their adjacent sequences, meet the above requirement.

### Gene expression analysis

Raw reads were trimmed by Skewer (v0.2.2)^39^ to remove adapter sequences and then aligned against the reference genome (GRCh37/hg19) by STAR (v2.4.2a)^40^. RSEM (1.2.29)^41^ were used to perform expression abundance quantification based on the uniquely mapped reads. Gene annotation GENCODE v19 was used in the above process.

### Somatic mutation calling

Potential somatic SNVs and small insertions or deletions (indels) were both called by MutTect2 in GATK4^42^ using default parameters based on paired-alignment files (tumor and normal bam files). SNVs were filtered with supported reads ≥ 4(≤2) and coverage ≥10 in tumor and (normal tissue), whereas indels were filtered with supported reads ≥5 (≤1) and coverage ≥10 in tumor and normal tissue. Moreover, somatic mutations and indels were annotated by Oncotator^43^.

### Identification of *BRCA1/2* variants

Somatic mutations (SNVs and indels) of *BRCA1/2* were derived from the results identified by MuTect2. We only retained somatic mutations assigned as non-stop, nonsense, splicing-site, and frame-shift for further study. Germline SNVs were called by Platypus^44^ with default parameters and then filtered with at least 30x coverage and VAF **>**8%. SNVs with a minor allele frequency> 1% in either the Exome Variant Server (EVS; http://evs.gs.washington.edu/EVS/) or the ExAC normal population cohort^45^ were removed. Germline deleterious SNVs of *BRCA1/2* were selected with annotating be pathogenic in the ClinVar database. Germline indels of *BRCA1/2* were called by SvABA^20^ using default parameters. Finally, somatic mutations (SNVs and indels) and germline mutations (SNVs and indels) of *BRCA1/2* were combined to be used in next association analysis.

### Mutational signatures

We applied the R package MutationalPatterns^46^ to estimate the contributions of 30 mutational signatures documented by the COSMIC for each sample. This package uses a well-founded NNLS (non-negative least-squares) algorithm from package Pracma (https://CRAN.R-project.org/package=pracma) to minimize the Euclidean norm of residual between COSMIC signature matrix (dot product with signature contribution) and count vector of 96 trinucleotide changes for each sample.

### Copy number calling

We estimated copy number profiling over 10 kb windows by using Patchwork^47^, and then calculated the normalized ratio of standardized average depth between normal tissue and tumor tissue. The purity and ploidy of each tumor were calculated on the local copy number of each segment and the allele frequency of each somatic SNV using ABSOLUTE1.0^48^.

### Somatic SV detection and validation

We applied SvABA^20^ and Delly^21^ to predict somatic SVs and their breakpoints using the suggested parameters. Recurrent SVs of different individuals from SvABA are regarded as germline events. SVs with Q value less than 10 are filtered. Delly-private SVs are manually checked. Of which SV that associate with copy number change are kept and merged into the SvABA cohort as the final cohort. Tumor samples from three patients are re-sequenced. The validation accuracy for these three patients is 93.25%, 90.33%, and 78.14%. Copy number changes from the patient with low accuracy exhibit intratumoral heterogeneity(Supplementary Fig. 3)

### Intratumor heterogeneity

If the genome varies greatly between different tumor regions of the same patient, we believe that the patient has high intratumor heterogeneity.

### The inference of microhomology and repair mechanism

Microhomology is identified for each SV by SVABA or Delly. The repair mechanism is inferred based on the microhomology length of each SV. We used criteria described by Lixing Yang^49^:NAHR: breakpoints have >100 bp microhomology; alt-EJ: breakpoints have 2 to 100 bp microhomology; NHEJ: breakpoints have 0–1 bp microhomology or 1–10 bp insertion; FoSTeS/MMBIR: breakpoints have >10 bp insertion

### Inference of kataegis

We inferred kataegis by using R package SeqKat. Kataegis is a pattern of SV occurring in the region which is enriched in somatic SNVs. These SNVs are mainly catalyzed by the AID/APOBEC family of proteins (C **>** T and C **>** G mutations).

### Identifying SV hotspots

We used an approach described by Glodzik^23^ to identify SV hotspots and using the PCF algorithm to determine genomic regions exhibiting rearrangement density much higher than that observed in neighboring genomic regions. We applied the PCF method to three categories of TDs to explore regions with a rearrangement density exceedingly twice the whole-genome background density and involving a minimum of 15 samples.

### TDP classification

We used a method described by Menghi^25^ to calculate a TDP score for each ESCC. For each tumor sample, we counted the total number of TDs and compared the observed (Obs_i_) and expected (Exp_i_) numbers of TDs for each chromosome,

1 $${{{{{\rm{i}}}}}}\!:{{{{{\bf{TDPscore}}}}}}=-\frac{{\sum }_{{{{{{\bf{i}}}}}}}\left|{{{{{{\bf{Obs}}}}}}}_{{{{{{\bf{i}}}}}}}-{{{{{{\bf{Exp}}}}}}}_{{{{{{\bf{i}}}}}}}\right|}{{{{{{\bf{TD}}}}}}}.$$

### Definition of BRCAness

BRCAness signature is the phenotype of *BRCA1* or *BRCA2* mutations, and it describes the situation in which an HRR(Homologous Recombination Repair) defect exists in a tumor in the absence of a germline *BRCA1* or *BRCA2* mutation.

### Complex rearrangement reconstruction

Complex rearrangements refer to events that involve four or more breakpoints. Breakpoints in these events are thought to occur simultaneously and phased into a new genomic configuration^50^. The connection of breakpoints could be imagined as a graph and path through four or more breakpoints are potential complex events or a sub-path of long, complex events. Next, we integrated the copy number of breakpoints and applied DFS (Depth-First search) to the graph to get potential complex events. The complex events are further refined and classified. In detail, there are five steps to identify complex rearrangements.

1. **Graph construction**. Each breakpoint is treated as a node. There is two class of edge: sequence edge and SV edge. SV edge refers to two breakpoints from the same SV; sequence edge refers to a connection between breakpoints that from distinct SVs. Thus, two breakpoints connected by a sequence edge are from the same chromosome region and generally near to each other (<5 Mb). To reduce the complexity of the graph, each breakpoint has at most two sequence edges. In addition, the orientation of the breakpoint end from a sequence edge is appropriate to form an edge. Simple TD and deletion within which no other breakpoints are released before graph construction to remove the influence of simple SVs on graph construction.
2. **Computation of copy number of breakpoints and sequence edge**. Complex events generally lead to copy number changes between nearby (left and right) genomic region of a breakpoint and copy number gain of a sequence edge. Thus, normalized coverage of genomic locus adjacent to breakpoint and sequence edge is computed for further graph refinement.
3. **Identification of complex rearrangements**. There are two manners to identify complex rearrangements. One method is based on anchored breakpoints. In other words, two anchored breakpoints are used as start and end breakpoints. Paths connected to these two breakpoints are potential complex events. Another manner is based on DFS, which could find all potential paths. We found anchor-based methods are suitable for bridge deletion, cycles of TD, unbalanced translocations, and cycles of templated sequences. Two anchored breakpoints are neighbors in genome positions. DFS-based manner is more suitable for unbalanced inversions identification. It is also helpful in the prediction of ecDNA and chromothripsis events.
4. **Refinement of complex rearrangements**. For bridge deletion and cycles of TD, the normalized coverage of anchored breakpoints is lower or higher than locally non-affected region, respectively. Besides, coverage of sequenced edge involved in bridge deletion or cycles of TDs is higher than nearby non-affected region. For complex unbalanced translocations, anchored breakpoints are first searched based on whether the breakpoints could divide the chromosomal arm into two parts with distinct copy number baseline. After that, graph paths that connect two anchored breakpoints are identified and filtered path within which the coverage of sequence edge is not appropriate. For unbalanced inversions, we require that there exists copy number loss within the affected region, while sequence edge within unbalanced inversion did not show copy number change.
5. **Visualization of complex events**. The coverage of complex events is further visualized as an option for manual inspection. It is beneficial for chromothripsis inference, which usually demonstrates several sporadic genomic paths in our analyses due to incomplete SV sets.

### Comparison to other methods for complex rearrangement identification

We summarized three studies^14,51,52^ that attempt to untangle the complex rearrangements. GRIDSS2^51^ employs the single breakend assembly, which helps phase nearby SVs and identify rearrangements caused by SINE Alu, LINE L1HS insertions, or rearrangements involving centromeric sequence. Li et al.^14^ classify and annotates complex SVs into several types: Local-distant cluster, Local n-jump, and Cycle of templated insertions. Starfish^52^ is a tool that integrates copy number profiles and SV clusters to identify subtypes that did not aim for specific complex rearrangement. Compared to other tools, our method is based on the simple idea that complex rearrangement is thought of as a combination of a simple SV and cycles of TSIs. Based on this idea, it could identify four types of complex rearrangements (unbalanced inversion and translocation, bridge deletion, cycles of TDs) that are curated in literature. Some of these rearrangements partially overlap with results from GRIDSS2 and Li et al., but not exact. In addition, it could nominate TSI-mediated fold-back inversion, which GRIDSS2 and Li et al. miss. (Supplementary Table 2).

### BFB inference

We inferred BFB events by detecting fold-back inversion and telomere loss which is introduced by Campbell^32^. Fold-back inversions were detected based on three criteria: (1) the single inversions were without reciprocal support-read clusters, (2) the inversion caused a copy number change (*q* **<** 0.001), and (3) the two ends of the breakpoints had to be separated by less than 100 kb.

### Chromothripsis

Chromothripsis often involves tens of SVs that occur simultaneously^18^. Owing to the drawback of short sequencing, it is not easy to get the complete SV set of a chromothripsis^53^. Complex rearrangements with eight or more breakpoints are manually inspected for chromothripsis events in our data. Copy numbers of segments oscillate between 2 or three copy number states are identified as potential chromothripsis events. The distribution of SV types is uniform within affected regions

### Validation of the prognostic ability of TSI fold-back inversions

To validate the prognostic ability of TSI fold-back inversions, we constructed a gene expression signature for TSI fold-back inversions using 133 tumors with available RNA-seq data. We first applied limma^54^ with default parameters to identify differentially expressed genes (DEGs). The final 598 DEGs were defined at a threshold of a 2-fold change and *p*-value < 0.05. Correction for multiple hypothesis testing was not performed for these DEGs. A random forest classifier was generated to detect ESCC with complex fold-back inversions by using randomForest R package based on the identified DEGs. Ten repeats of 10-fold cross-validation in our data set led to the optimal selection of 185 DEGs that demonstrated the lowest classification error and an area under receiver operating curve (AUC) of 83.07%. The AUC curves were drawn using pROC R package. The classifier was then applied in another independent 119-ESCCs cohort and showed that the predicted ESCCs with complex fold-back had a similar worse prognosis.

### ecDNA detection

ecDNA generally leads to high-level focal amplifications that involve one or multiple amplicons. It also has a circular genomic structure. We firstly identify amplicons based on discontinuous high-level amplified regions (CN >= 5). Precisely, amplifications that could be connected by the SVs are thought to be in the same amplicon. Secondly, we employed AmpliconArchitect for these high-level amplicons and require that there exist a circular structure (also called simple cycle) within them. Finally, high-level amplified regions enriched for fold-back inversion (> = 2) are defined as BFB instead of ecDNA.

### Enrichment of super-enhancer elements

The E079 DNase-seq and H3K27ac ChIP-seq data were downloaded from the NIH Roadmap Epigenomics Mapping Consortium (http://egg2.wustl.edu/roadmap) and the KYSE180 H3K27ac ChIP-seq data were downloaded from GEO^26^.

### Immunohistochemistry

PTHLH antibody (ab197358, Abcam, Cambridge, UK) (1:200 dilution) was used as the primary antibody. The experimental steps were carried out according to the conventional step. Tissue microarrays (TMAs) of 134 ESCC and control tissues were hand-made by TMA manufacturing machine. TMA scanner is Pannoramic MIDI by 3D HISTECH manufactured. And after the TMAs scanning were completed, H Score (Histochemistry Score) were analyzed by Quant Center.

### Fluorescence in situ hybridization

To detect extrachromosomal oncogene amplification statue in esophageal squamous cell carcinoma, we performed FISH (fluorescence in situ hybridization) assay using FISH probe kit for detection of *EGFR, MYC* and *CCND1* amplification (Lot: FD-F0010, Guangzhou Exon Company; LBP F. 01006 and LBP F.01023, Guangzhou Anbiping Company; China). The experimental steps were carried out according to the kit instructions. The results of interpretation are according to the following way. First, tumor areas were identified on HE sections; Then, the same histocyte structures were found on the FISH specimens as on the HE stained sections. We would count fluorescent signal points on at least 20 infiltrating cancer cells using fluorescence microscopy.

### PCR-Sanger sequencing validation

For validation of SV events, we performed sanger sequencing assay on tumor and matched normal tissues from gDNA from FFPE sample were purified using Gene ReadTM DNA FFPE kit (QIAGEN, Germany). PCR product was analyzed by agarose gel electrophoresis. Amplified PCR products were gel purified and then sequenced via the Sanger method. The primers are listed in Supplementary Table 3.

### Cell lines and cell culture

ESCC cell lines (KYSE150, KYSE180, and KYSE450) used in the research were purchased from Cell Bank of Type Culture Collection of Chinese Academy of Sciences. All of the cells were authenticated by short tandem repeat (STR) analysis. All cell lines were routinely tested to ensure they are free of mycoplasma contamination (VenorTMGeM Mycoplasma Detection Kit, Sigma-Aldrich).

The ESCC cell lines were cultured in RPMI-1640 medium supplementary (Hyclone) with 10% fetal bovine serum (Gibco), 100 U/ml penicillin, and 100 μg/ml streptomycin. All cells were tested for mycoplasma contamination and cultured at 37 °C in a humidified atmosphere with 5% CO_2_. According to the cell state, the cell culture medium was replaced. When the cells fusion was about 80–90%, the cells were subcultured.

### Luciferase reporter assays

We downloaded DNaseseq data and H3K27ac chromatin immunoprecipitation (ChIP)- seq data^26^ and identified eight enhancers, denoted e1 to e8 here, in the 200 kb regions downstream of *PTHLH*. Luciferase reporter assays were performed as follows. Individual enhancer regions were cloned upstream of the pGL4.10-E4TATA promoter vector using KpnI and XhoI restriction enzyme sites. The reporter constructs were co-transfected with a control Renilla luciferase constructed into KYSE150 and KYSE450 cells. After 48 h, the cells were collected with ice-cold PBS and harvested in the reported lysis buffer. Ten μl of the supernatants were mixed with 50 μl of solution I and measured for firefly luciferase activity by using a TransDetect double luciferase reporter assay kit (Transgene, China). Then mixed with 50 μl solution II and measured for Renilla luciferase activity. All reactions were performed in triplicate. Primers used for cloning are listed in Supplementary Table 3.

### CRISPR/Cas9-mediated deletion of the enhancer region

All sgRNA sequences are listed in Supplementary Table 4. sgRNAs were cloned into LV-sgCas9-P2A-puro (Shanghai Genechem Co., Ltd.). Vectors were transfected into KYSE450 cells and were selected with puromycin (Invitrogen, Carlsbad, CA, USA) for 2 weeks. To detect deletion of the e3 and e5 enhancers, genomic DNA was first extracted and then used for PCR using the PrimeSTAR GXL DNA Polymerase (Takara, Japan) with the primers listed in Supplementary Table 4.

### Real-time quantitative PCR (qPCR)

qPCR was used for measuring mRNA expression. Total RNA was isolated from cells using the RNA extraction reagent (RNAiso Plus, Takara, Bio Inc, Japan). Reverse transcription was performed using PrimeScriptTM RT reagent kit (Takara, Bio Inc, Japan), and qPCR was performed using the SYBR Green Premix Ex TaqTM (Takara, Bio Inc, Japan). The primer of *PTHLH* is in Supplementary Table 3. All qPCR reactions were performed in triplicate with an Applied Biosystems StepOnePlus (ABI, Foster City, CA, USA). The relative expression of genes was determined by normalization to GAPDH expression according to the manufacturer’s instructions. Data analysis was performed using the formula: 2^−ΔΔCt^.

### Transfection and cell-proliferation assays

The siRNA (RiboBio, Guangzhou, China) of *PTHLH* was used to knockdown the *PTHLH* gene in KYSE150 and KYSE180 cells. The siRNA sequences are: si-*PTHLH*-RNA1: 5′-ACTGCTTTATACTTTGTCA-3′;si-*PTHLH*-RNA2: AATGGCAAATAGTCTTGT A-3′; The siRNA was transfected with riboFectTM CP Transfection Kit (C10511-1). Cell proliferation assay was performed by MTT and Colony-forming. MTT assay was performed as follows. 5000 cells per well were plated into 96-well plates and cultured at the normal condition for 24 h, 48 h, 72 h and 96 h, respectively. Then 20 μl of 5 mg/ml of MTT (Invitrogen) was added to each well and cultured until crystals were formed at 37 °C. After 4 h, 200 μl DMSO was used to dissolve the crystals and measured the absorbance at 490 nm. The DMSO-treated be seen as a control. The colony-forming assay was performed as follows. 800 cells per well were seeded in 6-well plates and cultured in RPMI-1640 with 10% FBS at 37 °C in a humidified atmosphere with 5% CO_2_ for 10 days. In the end, the colonies were fixed in 4% paraformaldehyde for 30 min and stained with 1% crystal violet for 20 min at room temperature. The colonies containing more than 50 cells were photographed and counted. All experiments were independently repeated thrice.

### Migration and invasion assays

Transwell migration and invasion assays were performed as follows. 1.2 × 10^5^ cells were seeded into the upper chamber of a 24-well plate and cultured with FBS freed RPMI-1640 medium. The lower chambers were filled with 600 μl RPMI-1640 with 10% FBS. The plate was incubated at 37 °C in a humidified atmosphere with 5% CO_2_ for 48 h. Then the cells that passed through the membrane were fixed with 4% formaldehyde and stained using 0.1% crystal violet. Random five fields were chosen to count the number of transmigrated cells. For the transwell invasion assays, the upper chambers were pre-coated with 100 μl of Matrigel (1: 6 mixed with FBS-free media; BD Biosciences, Heidelberg, Germany) and proceeded using the same as described above.

### Wound healing assay

Wound healing assay was performed as follows. 1 × 10^6^ cells per well were seeded in 6-well plates and cultured in RPMI-1640 with 10% FBS. When the cells reached 90% confluence, a scratch was created with a 200 μl pipette tip. The cells were cultured without FBS freed RPMI-1640 medium for 48 h. Micrographs were captured at 0 h, 24 h, and 48 h.

### Survival analysis

We used the log-rank test to perform survival analysis and plotted the survival time distribution by the Kaplan–Meier method in R package Survival. The multivariate surviving model were constructed by coxph function in R package Survival to compute the HR for putative factors.

### Quantification and statistical analysis

All statistical test was performed in R (version 4.0). The non-parametric Mann-Whitney U test, Fisher’s exact test, Student’s *t*-test, and one-way ANOVA test were used to compare groups. We also used the log-rank test to perform survival analysis. The association between factors was assessed by Spearman correlation. For all statistical tests used, we assumed that data are independent. Box plots show median values and middle quartiles. The random forest model was generated using the randomForest package. The ROC curves were drawn using pROC package.

### Reporting summary

Further information on research design is available in the Nature Research Reporting Summary linked to this article.

## Supplementary information

Supplementary Information

Description of Additional Supplementary Files

Supplementary Data 1

Supplementary Data 2

Supplementary Data 3

Supplementary Data 4

Supplementary Data 5

Supplementary Data 6

Supplementary Data 7

Reporting Summary

## Data Availability

The raw sequencing data generated in this study have been deposited in the Genome Sequence Archive (Genomics, Proteomics & Bioinformatics 2021) in National Genomics Data Center (Nucleic Acids Res 2022), China National Center for Bioinformation/Beijing Institute of Genomics, Chinese Academy of Sciences (GSA-Human): HRA003107 (WGS&RNA-seq, https://ngdc.cncb.ac.cn/gsa-human/browse/HRA003107), HRA000021 (WGS, https://ngdc.cncb.ac.cn/gsa-human/browse/HRA000021) and HRA002508 (WGS & Nanopore, https://ngdc.cncb.ac.cn/gsa-human/browse/HRA002508). The raw sequencing data are available under controlled access due to data privacy laws related to patient consent for data sharing and the data should be used for research purposes only. Access can be obtained by approval via their respective DAC (Data Access Committees) in the GSA-human database. According to the guidelines of GSA-human, all non-profit researchers are allowed access to the data and the Principle Investigator of any research group is allowed to apply for Controlled-access of the data. For data requests, please refer to the detailed guide: https://ngdc.cncb.ac.cn/gsa-human/document/GSA-Human_Request_Guide_for_Users_us.pdf. DAC will respond within two weeks. The data will be available within a week once the access has been granted and they will be available to download for one year. The human genome database used in this paper is version hg19 (https://hgdownload.soe.ucsc.edu/goldenPath/hg19/bigZips/). The publicly available RNA-seq data was downloaded from GEO database with accession number GSE53625^31^. The publicly available Chip-seq data used in this study are available in the GEO database under accession code GSE155187^26^. Source data are provided in this paper as a Source Data file. The remaining data are available within the Article, Supplementary Information, or Source Data file. Source data are provided with this paper.

## Source data

Source Data

## Code availability

The analysis code of SVs at https://github.com/ZHOUYong0530/FindRear.
