# Supplementary material for: Characterization of somatic structural variations in 528 Chinese individuals with Esophageal squamous cell carcinoma
Source: Nat Commun. 2022 Oct 22;13:6296. doi: 10.1038/s41467-022-33994-3 (PMC9588063; doi:10.1038/s41467-022-33994-3)
Supplement: Supplementary file 1 — Supplementary Information [file 41467_2022_33994_MOESM1_ESM.pdf]

Supplementary Figure 1

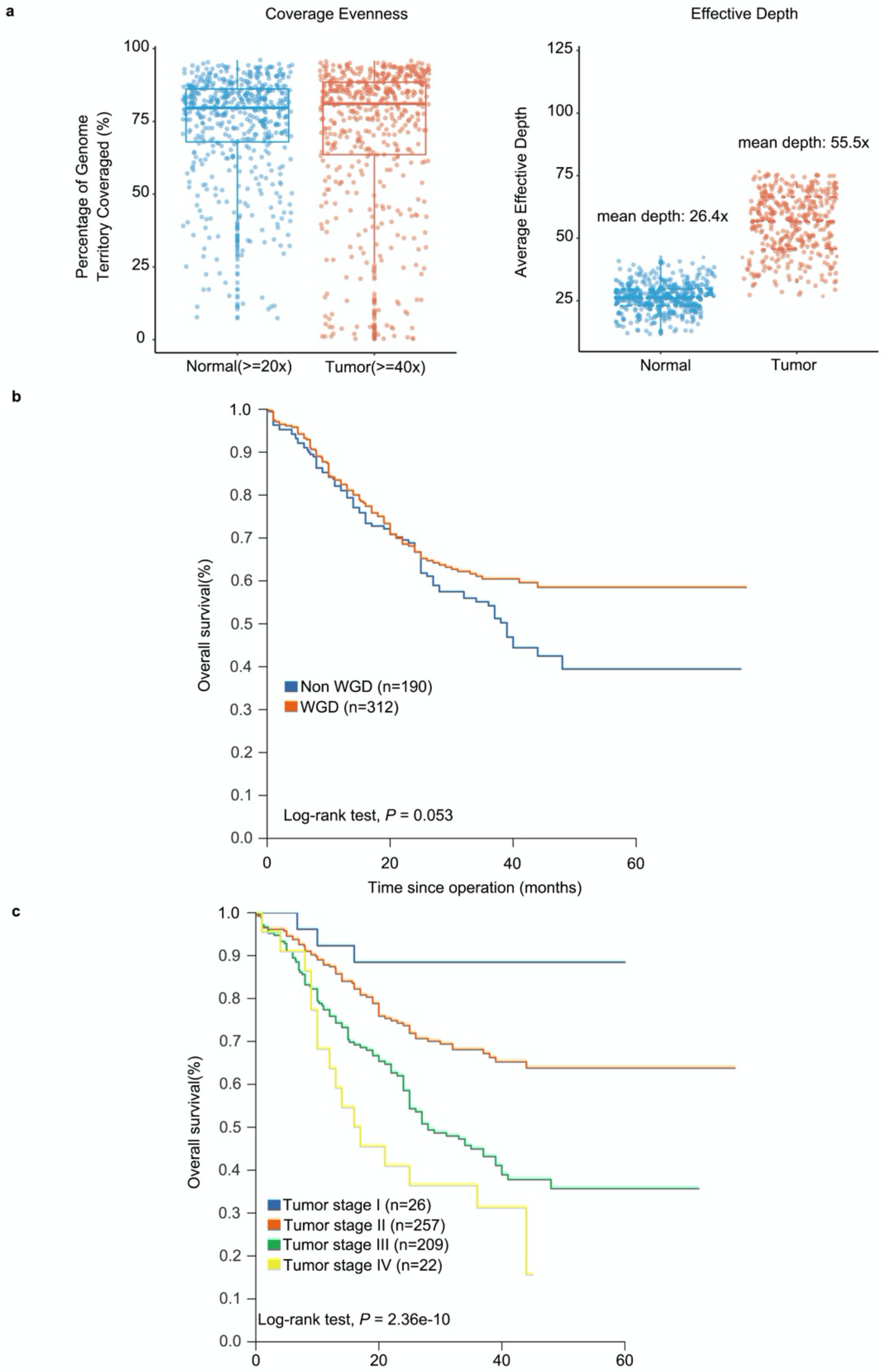

Supplementary Figure 1. Sequencing information of ESCCs. (a) Sequencing depth and normal of normal and tumor samples. n=528 biologically independent pairs of samples. Box plot data are presented as the median ± standard deviation. On the boxplots the horizontal line indicates the median, the box indicates the first to third quartile and whiskers indicate 1.5 × the interquartile range. **(b)** Kaplan-Meier survival curves show the survival outcome of WGD in ESCC. Statistical analysis

is performed with Log rank test. **(c)** Kaplan-Meier survival curves show the survival outcome of ESCCs different tumor stage. Statistical analysis is performed with Log rank test. Source data are provided as a Source Data file.

Supplementary Figure 2

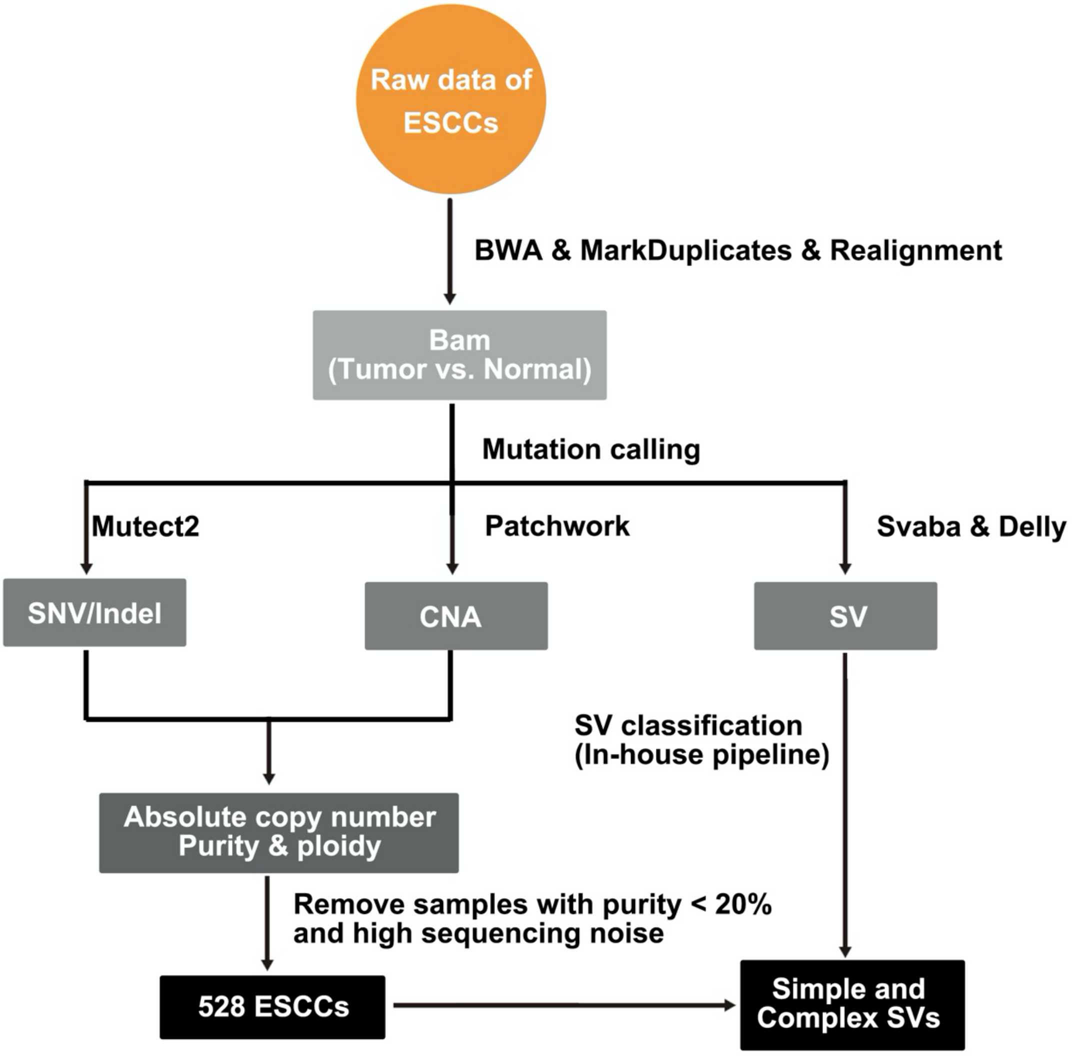

Supplementary Figure 2. A flow chart depicting the workflow used for identifying simple and complex SVs.

Supplementary Figure 3

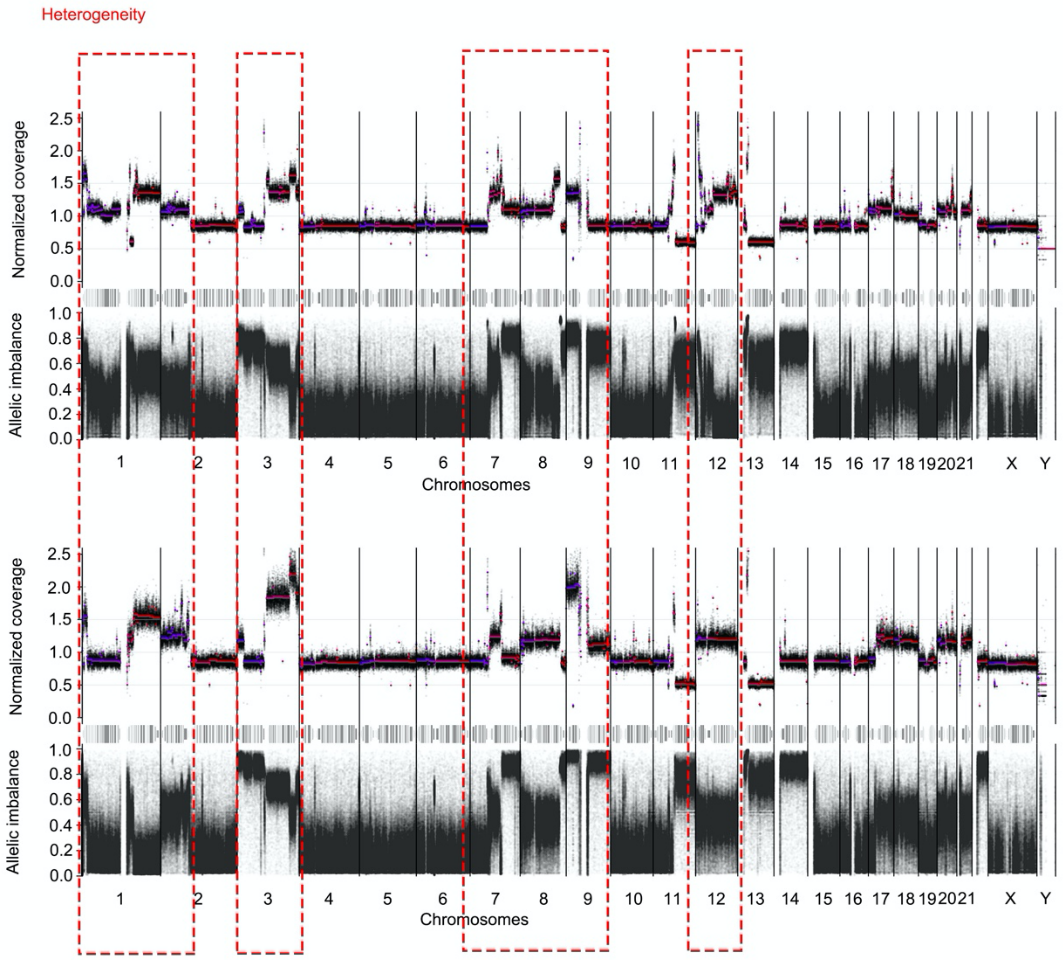

Supplementary Figure 3. The genome heterogeneity of copy number profiles of two samples from the same patient. Normalized coverage and allelic imbalance are shown. Red box highlighted the heterogeneity of these two samples. Source data are provided as a Source Data file.

Supplementary Figure 4

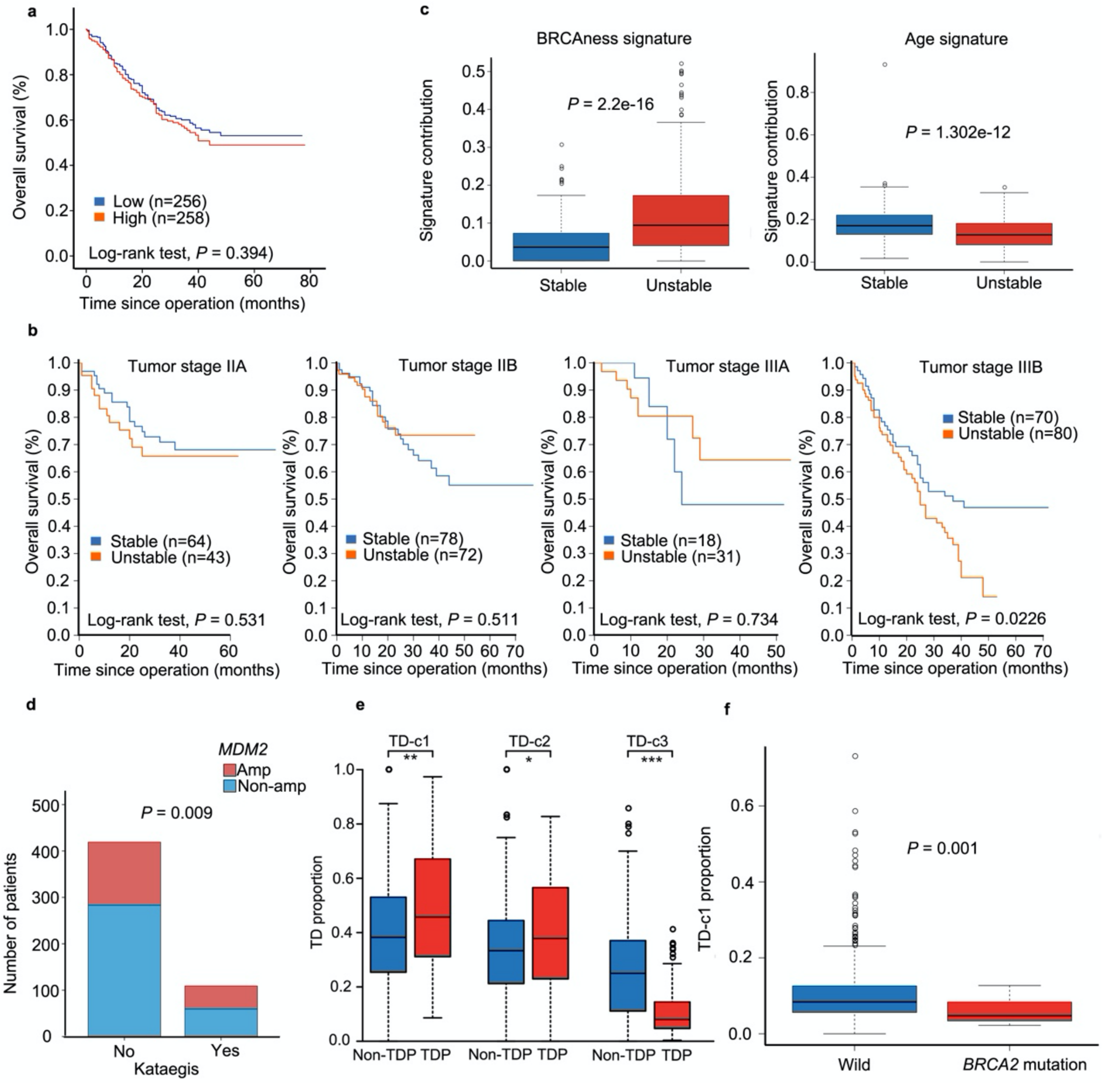

Supplementary Figure 4. (a) Kaplan-Meier survival curves show the survival outcomes of SV-burden in ESCC. **(b)** Kaplan-Meier survival curves show the survival outcomes of stability in ESCCs in different tumor stages. **(c)** Correlations of stability with Age and BRCA signatures. n=528 biologically independent samples. On the boxplots the horizontal line indicates the median, the box indicates the first to third quartile and whiskers indicate 1.5 × the interquartile range. **(d)** Bar plots show the association between focal amplification of *MDM2* with kataegis. **(e)** Boxplot shows the higher proportion of TD-c1,TD-c2 and lower proportion of TD-c3 in ESCCs with TDP. n=528 biologically independent pairs of samples. On the boxplots the horizontal line indicates the median, the box indicates the first to third quartile and whiskers indicate 1.5 × the interquartile range. **(f)** Box plot shows the lower proportion of TD-c1 in ESCCs with *BRCA2* mutation. On the boxplots

the horizontal line indicates the median, the box indicates the first to third quartile and whiskers indicate 1.5 × the interquartile range. n=528 biologically independent samples. Statistical analysis is performed with student-t test. *$P \leqslant 0.05$, **$P \leqslant 0.01$, ***$P \leqslant 0.001$. Source data are provided as a Source Data file.

Supplementary Figure 5

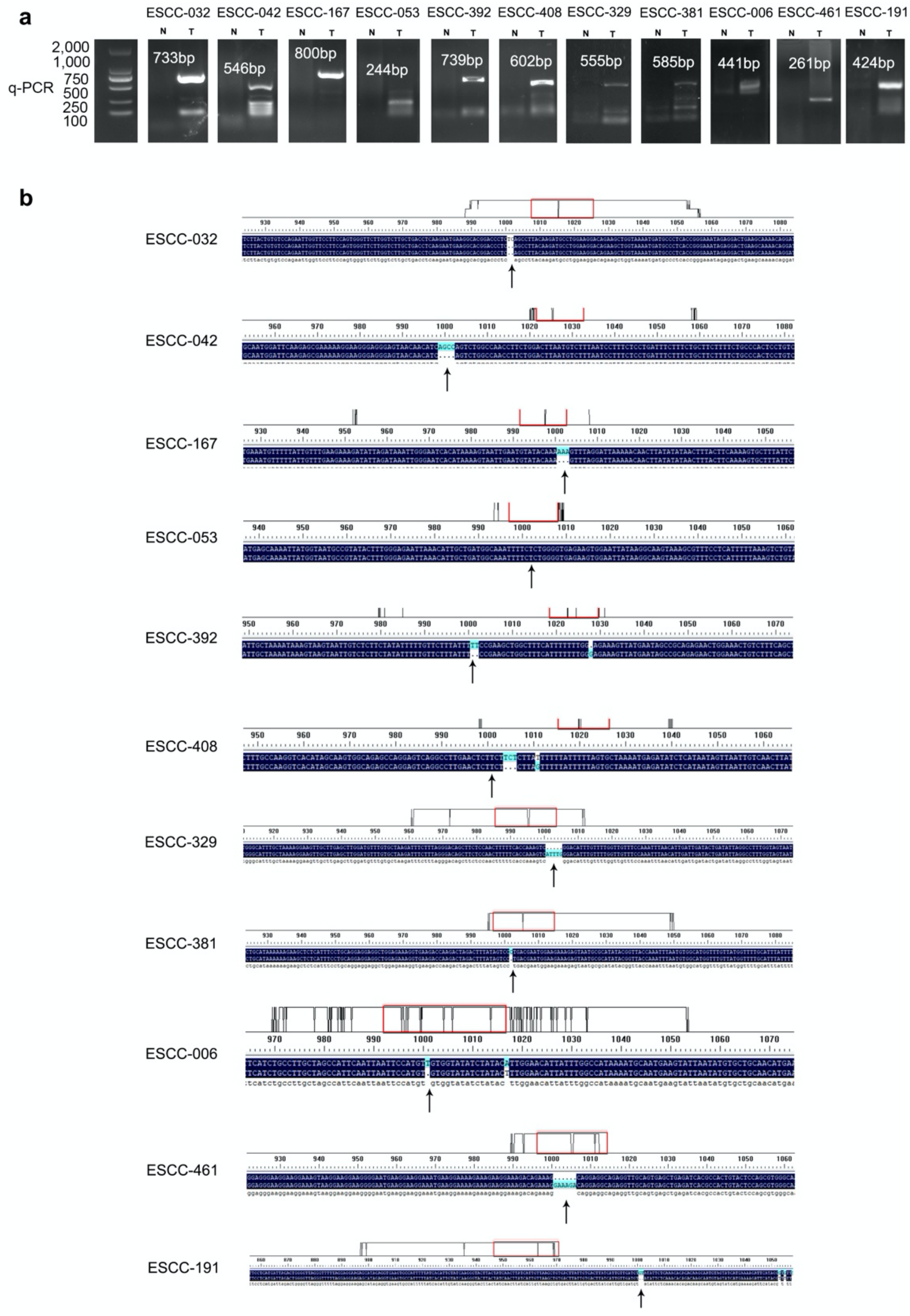

Supplementary Figure 5. Verification of TDs consisting of gene *PTHLH*. (a) Q-PCR validation of TDs in 11 samples. **(b)** Sanger sequencing results of TDs in 11 samples. Source data are provided as a Source Data file.

Supplementary Figure 6

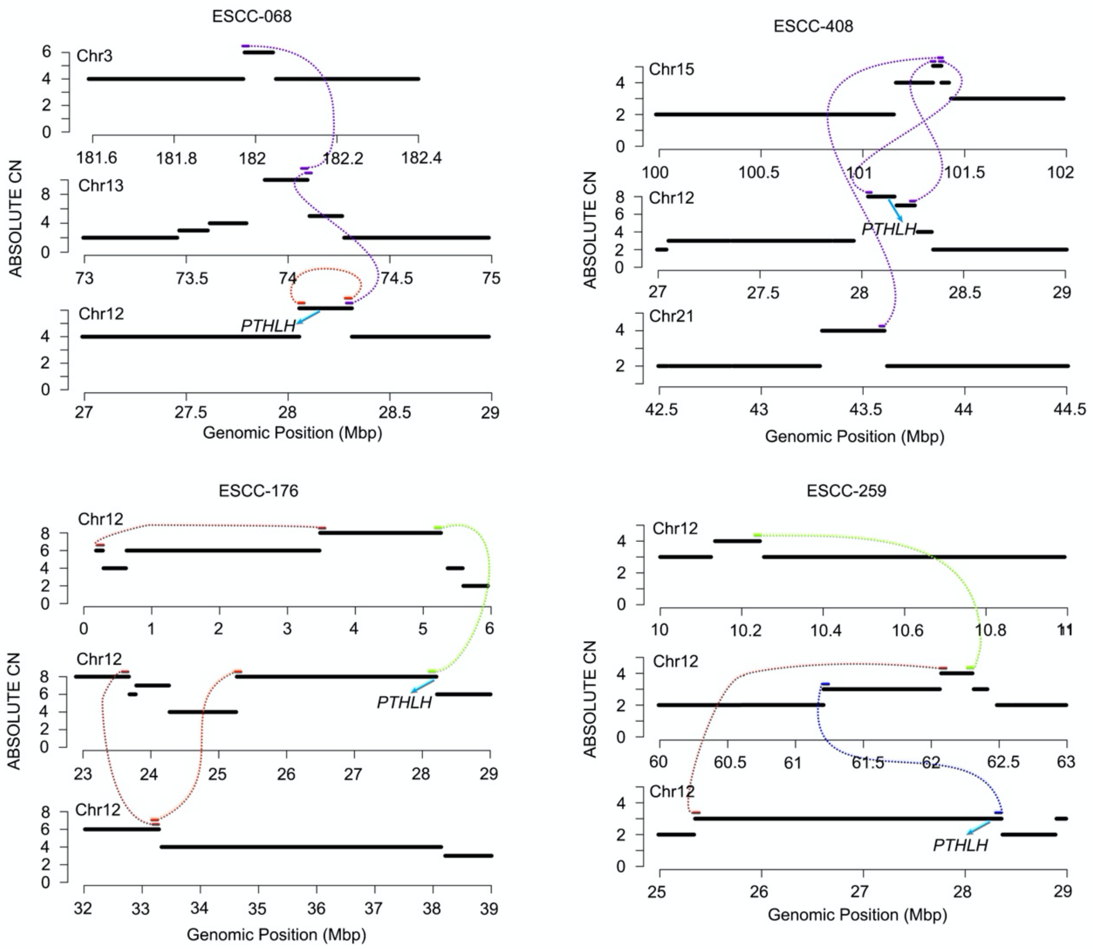

Supplementary Figure 6. A diagram of complex rearrangements causing *PHTLH* copy number gain. The black solid lines represent the copy number states of the target region, whereas structural variations are shown as colored dotted lines (blue: breakpoint strand "+-"; red: breakpoint strand "-+"; brown: breakpoint strand "--"; green: breakpoint strand "++"; purple: translocation) linking two segments. Source data are provided as a Source Data file.

Supplementary Figure 7

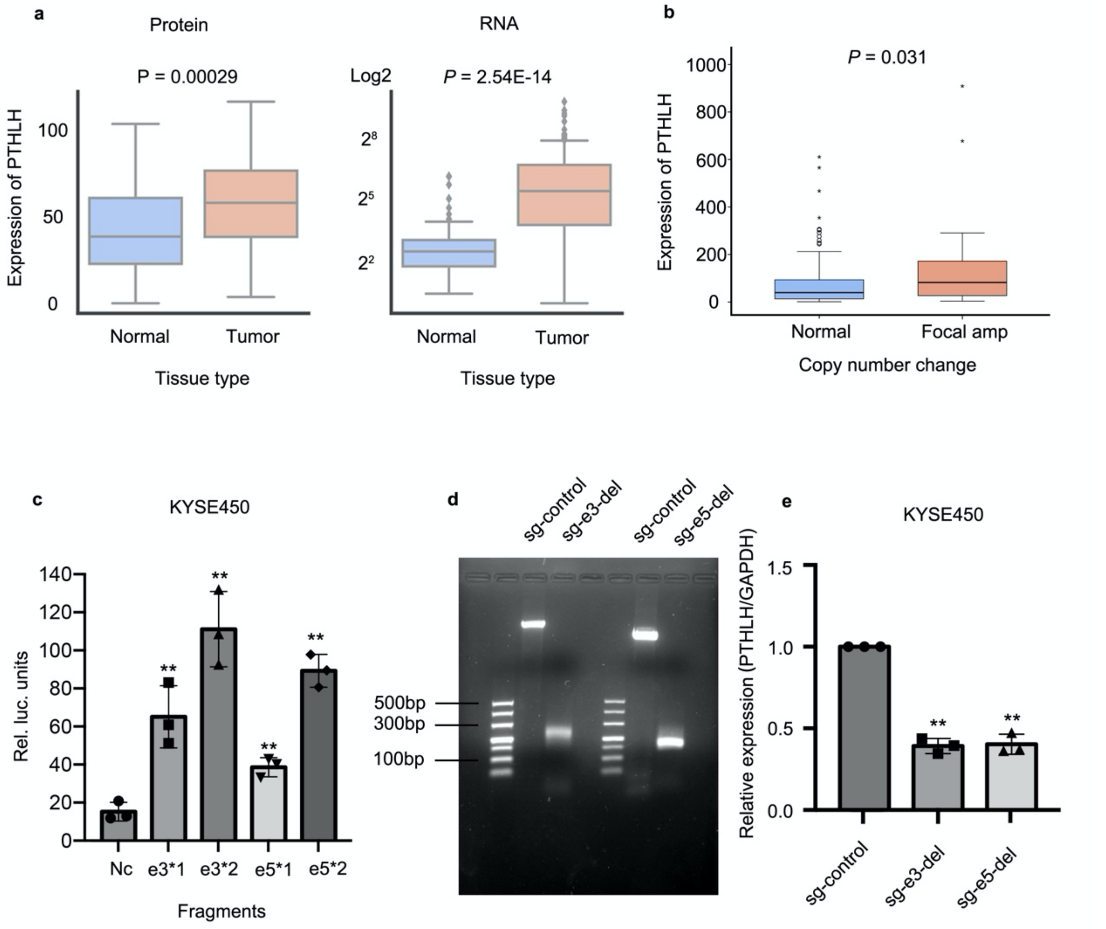

Supplementary Figure 7. *PTHLH* regulation function. (a) Box plot displays the PTHLH expression in tumor and normal. Left: RNA expression. Right: Protein expression. n=133 biologically independent samples. Statistical analysis is performed with student-t test. On the boxplots the horizontal line indicates the median, the box indicates the first to third quartile and whiskers indicate 1.5 × the interquartile range. (b) Box plot show the RNA expression of *PTHLH* between Focal amp samples and non-amp samples in ESCCs. Left: n=131 biologically independent pairs of samples. Right: n=133 biologically independent pairs of samples. Statistical analysis is performed with student-t test. On the boxplots the horizontal line indicates the median, the box indicates the first to third quartile and whiskers indicate 1.5 × the interquartile range. (c) Enhancer activity of duplicated e3 (e3 * 2) and e5 (e5 * 2) enhancers measured by luciferase-reporter assays in KYSE450. All bar-plot data are presented as the mean ± standard deviation**.** Three independent experiments were performed; each experiment was performed in triplicate. Statistical analysis is

performed with one-way ANOVA. (d) DNA electrophoresis results after knockout of e3 and e5 in KYSE450. Three independent experiments were performed. Statistical analysis is performed with one-way ANOVA. (e) Relative expression of *PTHLH* after knockout of e3 and e5 in KYSE450. All bar-plot data are presented as the mean ± standard deviation**.** Three independent experiments were performed; each experiment was performed in triplicate. Statistical analysis is performed with one-way ANOVA. *$P \leq 0.05$, **$P \leq 0.01$. Source data are provided as a Source Data file.

Supplementary Figure 8

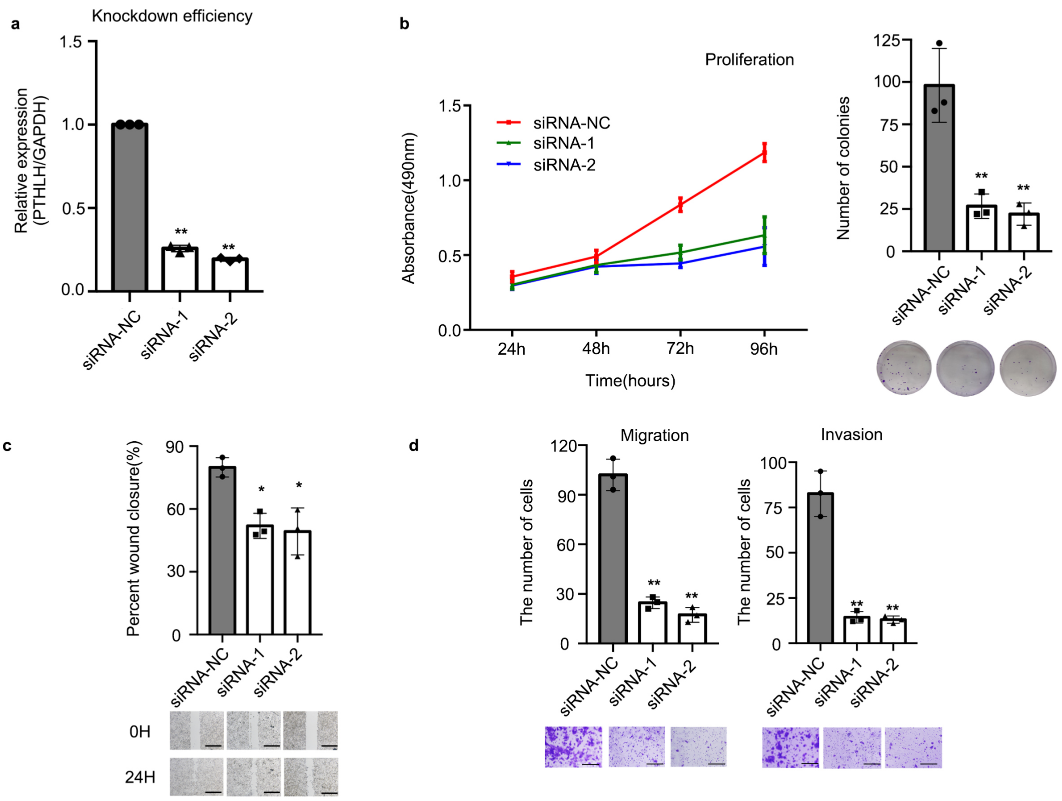

Supplementary Figure 8. Cell function experiment of *PTHLH* in ESCC cell line KYSE150. (a) Bar plot displays the knockdown efficiency of *PTHLH*. All bar-plot data are presented as the mean ± standard deviation**.** Three independent experiments were performed; each experiment was performed in triplicate. **(b)** Left panel: Line plot displays the proliferation of cells after *PTHLH* knockdown by MTT assay. Right panel: Bar plot displays the number of colonies in cells. All data are presented as the mean ± standard deviation**.** Three independent experiments were performed; each experiment was performed in triplicate. **(c)** Cell-migration monitored by wound healing assay and bar plot displays migration result. Scale bars, 500 μm. All bar-plot data are presented as the

mean ± standard deviation**.** Three independent experiments were performed; each experiment was performed in triplicate. (d) The transwell cell migration and invasion assay after PTHLH knockdown. All bar-plot data are presented as the mean ± standard deviation. Scale bars, 200 μm. Three independent experiments were performed; each experiment was performed in triplicate. Statistical analysis is performed with one-way ANOVA. *P ≤ 0.05, **P ≤ 0.01. Source data are provided as a Source Data file.

Supplementary Figure 9

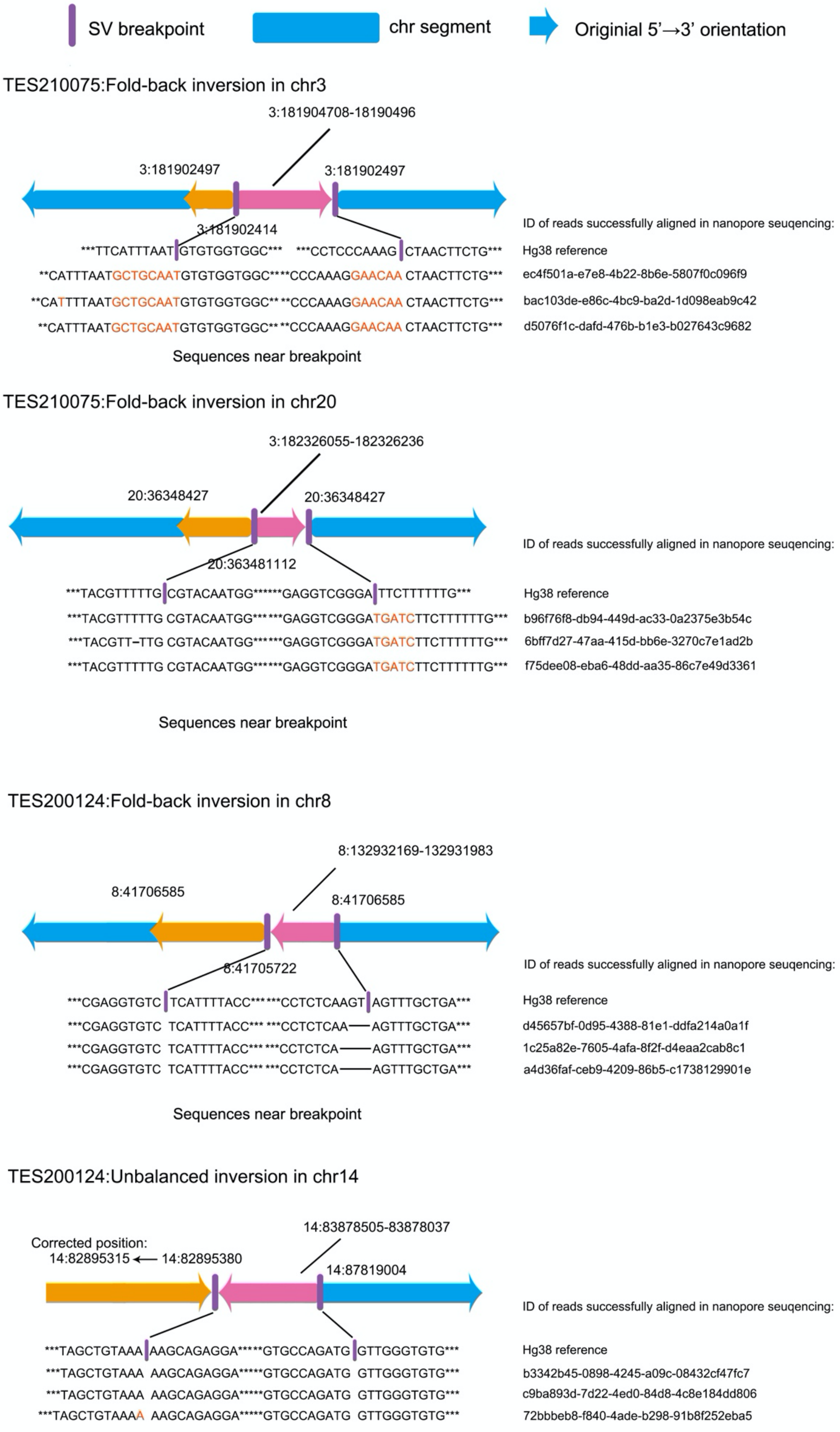

Supplementary Figure 9. **Schematic plot of four validated complex rearrangements in two ESCCs.** The upper part shows breakpoints of each complex rearrangement and derivative sequence; the bottom part displays representative aligned long-reads from the Nanopore sequencing. The nucleotide bases around the breakpoints are shown. Source data are provided as a Source Data file.

Supplementary Figure 10

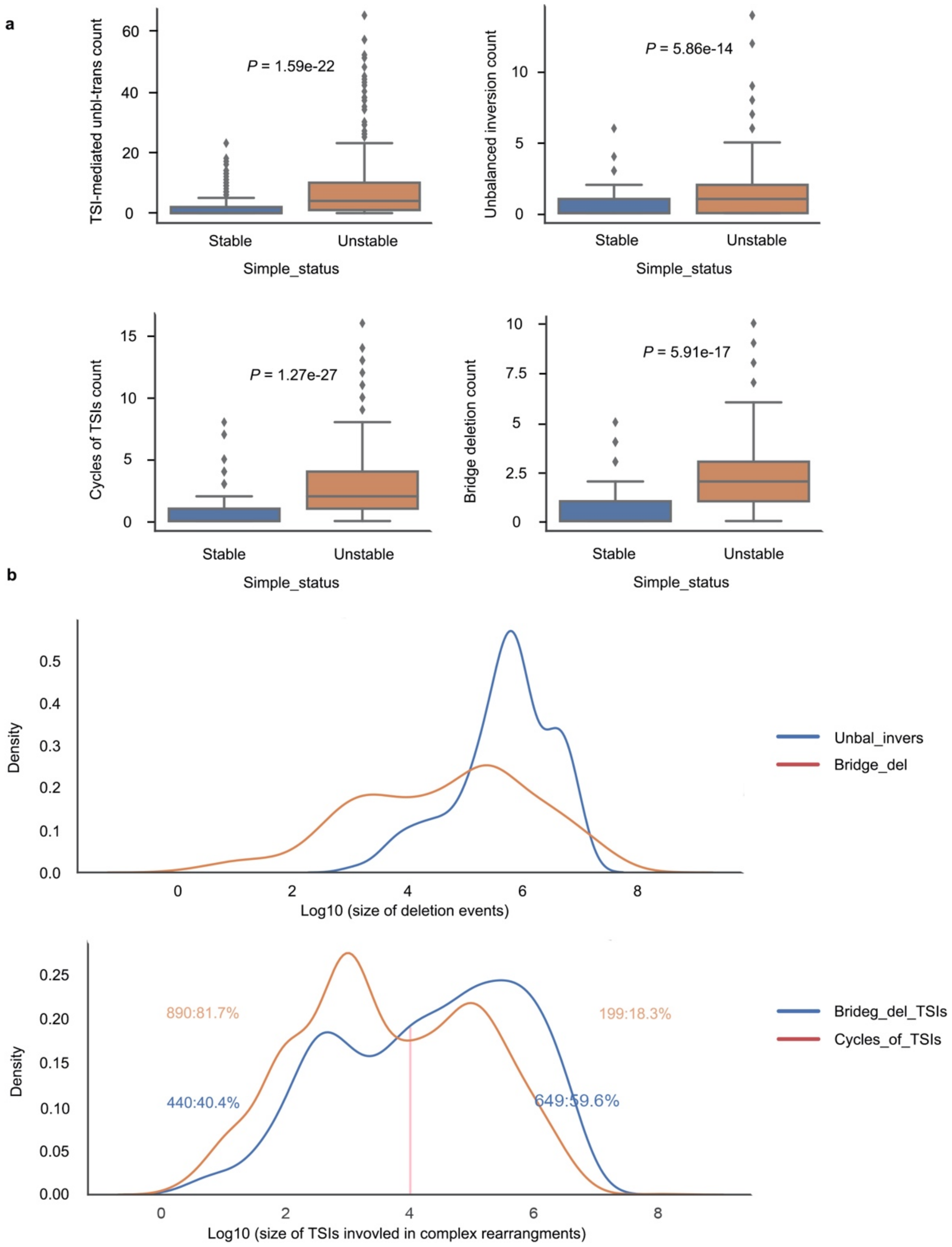

Supplementary Figure 10. **The genomic properties complex rearrangements**. **(a)** The box plots of complex rearrangements in stable and unstable group. n=528 biologically independent pairs of samples. On the boxplots the horizontal line indicates the median, the box indicates the first to third quartile and whiskers indicate 1.5 × the interquartile range. **(b)** The size of rearrangement and its TSI. Upper panel shows the size distribution of bridge deletion and unbalanced inversion; bottom panel shows the size of TSI for bridge deletion and cycles of TSIs, of which the percent of two groped TSI (classified more or less than 1kb) are marked. Statistical analysis is performed with student-t test. Source data are provided as a Source Data file.

Supplementary Figure 11

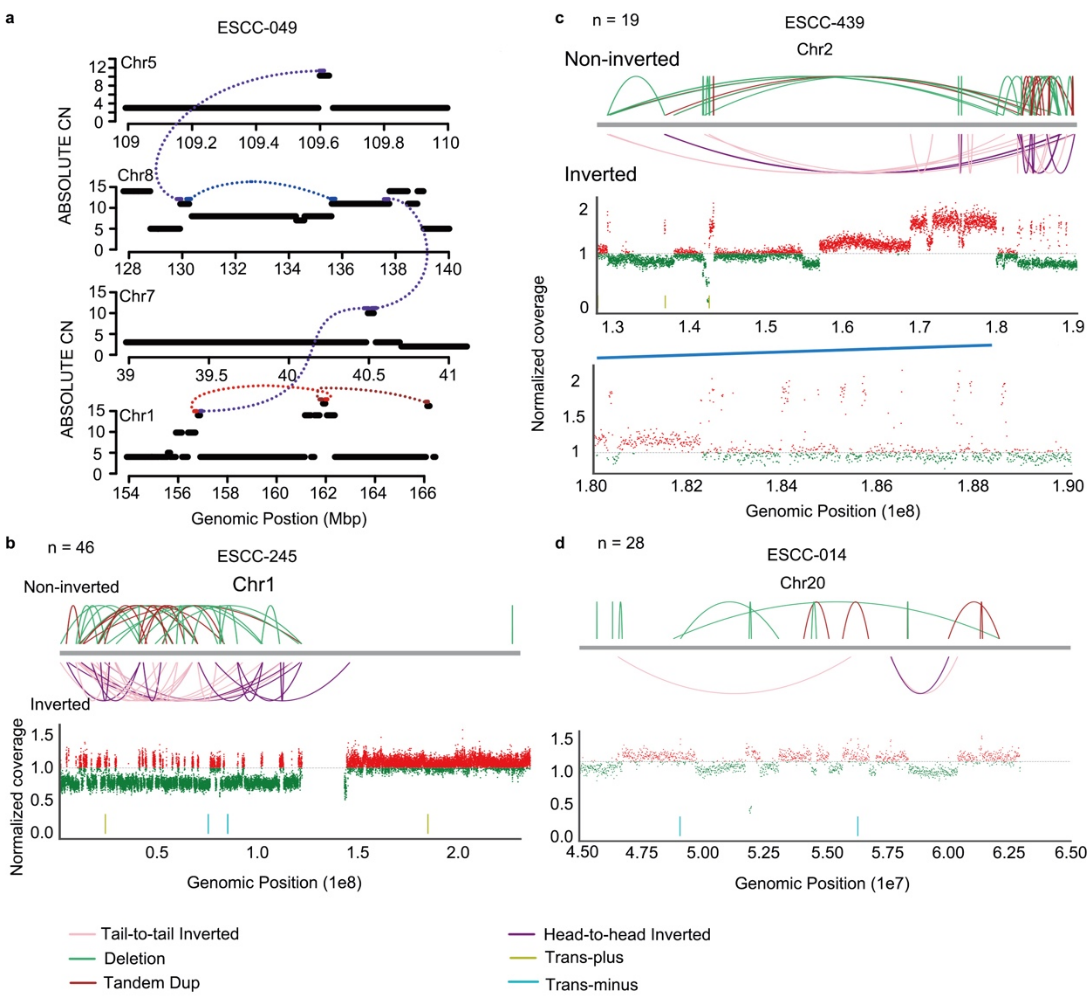

Supplementary Figure 11. Different types of chromothripsis in ESCCs. (a) A diagram of Inter-chromosomal chains. The normalized coverage and SV patterns of three types of chromothripsis is showed in **(b)**, **(c)** and **(d)** respectively. Of which grey line represents the median value of

normalized coverage. Each dot represents normalized coverage of 10kb and marked with red or green based on above or below the median value. Source data are provided as a Source Data file.

Supplementary Figure 12

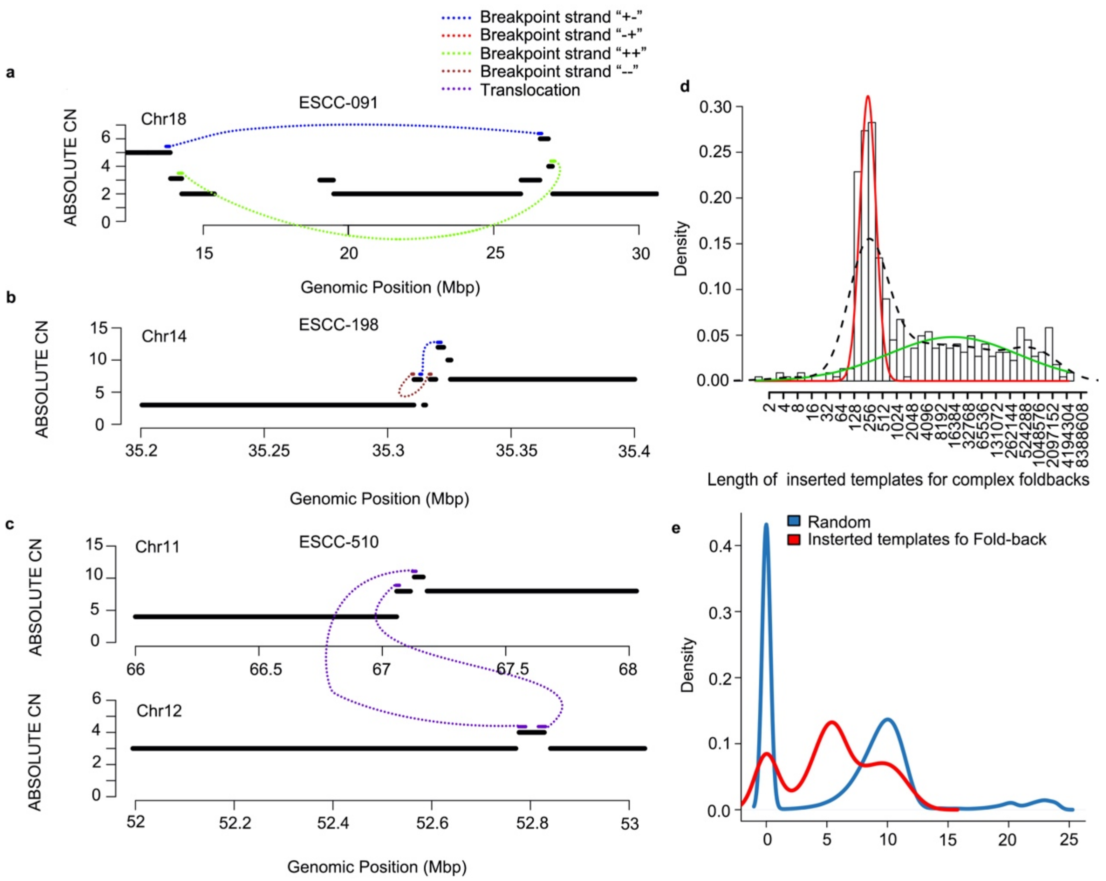

Supplementary Figure 12. Examples of TSI fold-back inversions. (a) Templated insertion from distal. **(b)** Templated insertion from local. **(c)** Templated insertion with translocation. The black solid lines represent the copy number states of the target region, whereas structural variations are shown as colored dotted lines (blue: breakpoint strand "+-"; red: breakpoint strand "-+"; brown: breakpoint strand "--"; green: breakpoint strand "++"; purple: translocation) linking two segments. **(d)** Density distribution of length of inserted templates for TSI fold-back inversion. **(e)** Density plot show inserted templates associated with Triplex mirror repeat. Source data are provided as a Source Data file.

Supplementary Figure 13

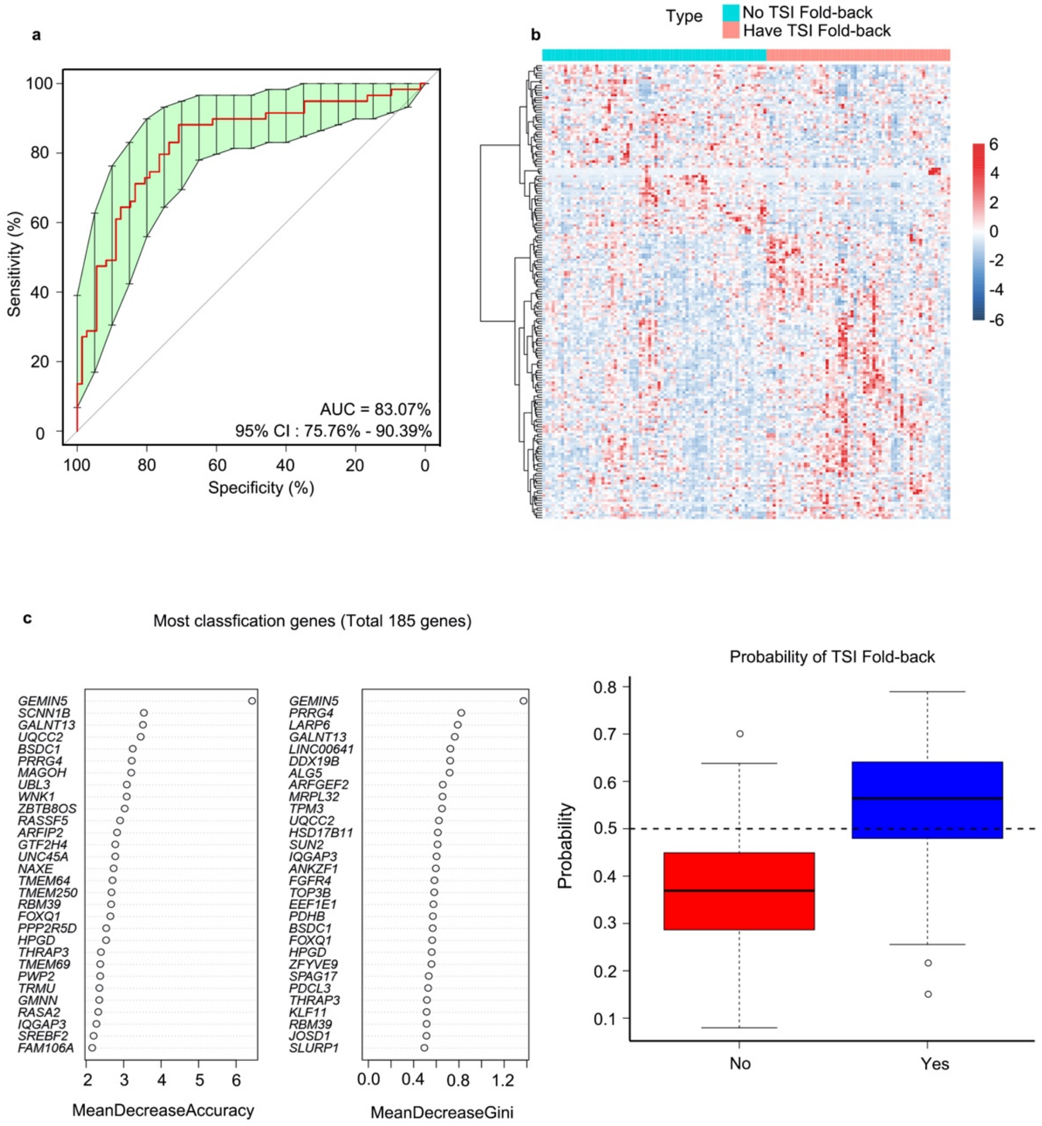

Supplementary Figure 13. Random forest classifier built from RNA-seq data. (a) Receiver operating characteristic (ROC) curves for DEGs between ESCCs with and without complex fold-back inversions by using available RNA-seq data. n=132 biologically independent pairs of samples. **(b)** Heatmap shows several DEGs between ESCCs with and without complex fold-back inversion. **(c)** Left: Plots show genes with most accuracy and Gini in the cross-validation dataset according to the random forest model. Right: Box-and-whisker plot for the probability of ESCC with complex fold-back inversions in the cross-validation dataset according to the random forest model. On the boxplots the horizontal line indicates the median, the box indicates the first to third quartile and whiskers indicate 1.5 × the interquartile range. n=131 biologically independent pairs of samples. Source data are provided as a Source Data file.

Supplementary Figure 14

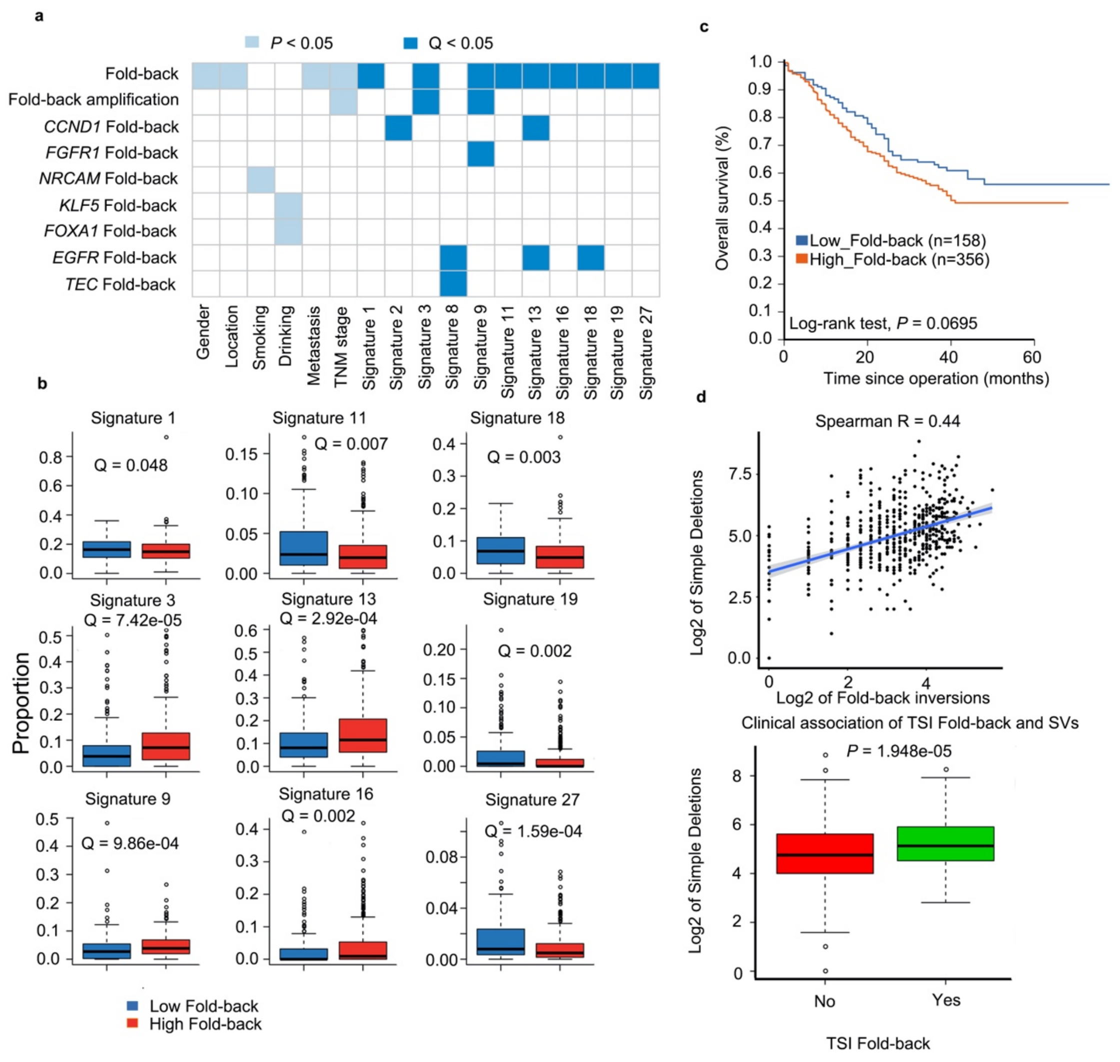

Supplementary Figure 14. Clinical significance of fold-back inversion. (a) Heatmap shows associations between different fold-back features and clinical factors. **(b)** Box plots show association between fold-back inversion and different signatures. n=528 biologically independent pairs of samples. On the boxplots the horizontal line indicates the median, the box indicates the first to third quartile and whiskers indicate 1.5 × the interquartile range. **(c)** Kaplan-Meier survival curves shows the survival outcomes of fold-back inversion. Statistical analysis is performed with Log rank test. **(d)** Up panel shows positive correlation between simple deletions and fold-back inversions. Bottom box plot shows association between simple deletions and TSI fold-back inversions. On the boxplots the horizontal line indicates the median, the box indicates the first to third quartile and whiskers indicate 1.5 × the interquartile range. n=528 biologically independent pairs of samples.

Statistical analysis is performed with student t test and multiple comparisons use corrected Q value**.** Source data are provided as a Source Data file.

Supplementary Figure 15

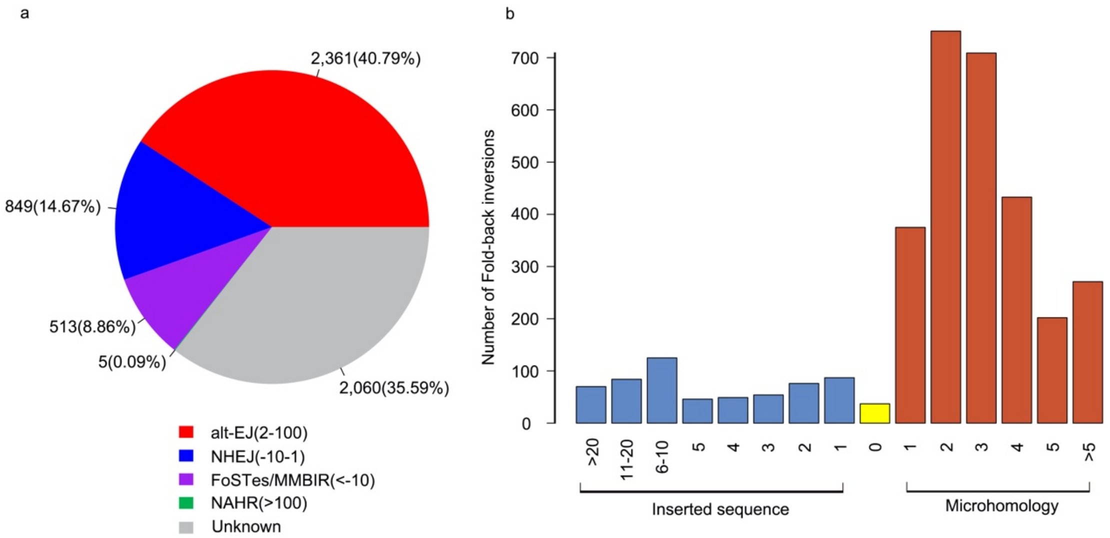

Supplementary Figure 15. Mechanisms of fold-back inversions. (a) Pie chart shows the frequencies of different mechanisms of fold-back inversions. **(b)** Patterns of micro-homology in fold-back inversions. Source data are provided as a Source Data file.

Supplementary Figure 16

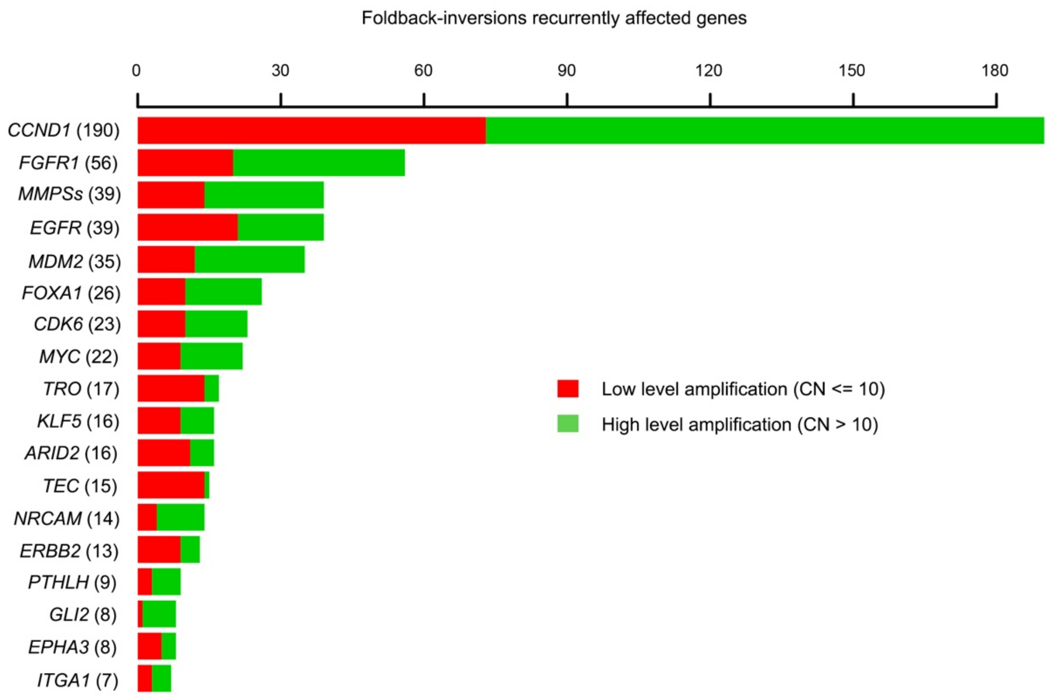

Supplementary Figure 16. Bar plot shows spectrums of fold- back inversions amplified oncogenes. Source data are provided as a Source Data file.

Supplementary Figure 17

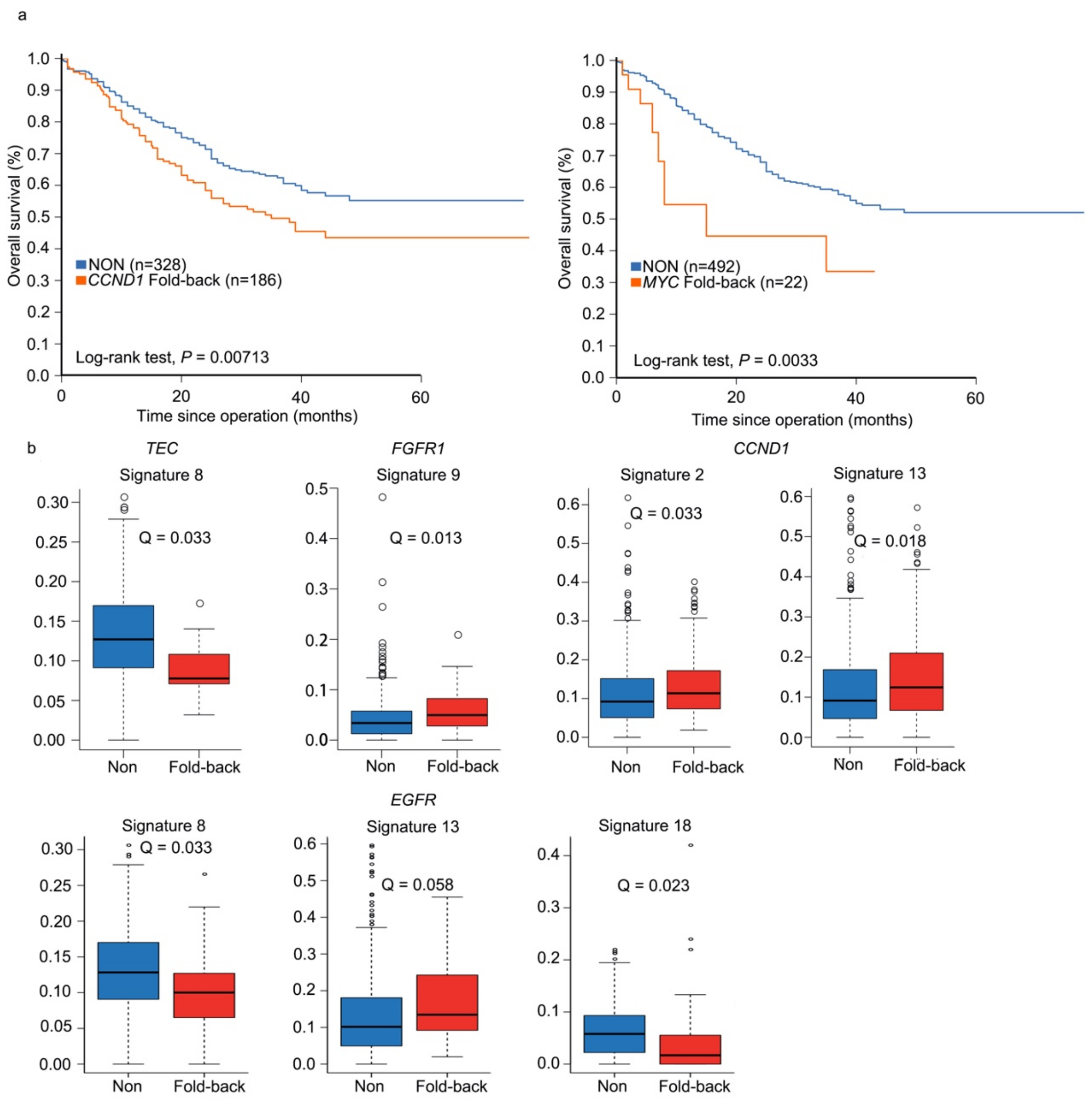

Supplementary Figure 17. Clinical significance of fold-back inversion with oncogenes. (a) Kaplan-Meier survival curves shows the survival outcomes of fold-back inversions with *CCND1* and *MYC*. Statistical analysis is performed with Log rank test. **(b)** Box plot show association between fold-back inversions with oncogenes and different signatures. On the boxplots the horizontal line indicates the median, the box indicates the first to third quartile and whiskers indicate 1.5 × the interquartile range. Statistical analysis is performed with student t test and multiple comparisons use corrected Q value. n=528 biologically independent pairs of samples. Source data are provided as a Source Data file.

Supplementary Figure 18

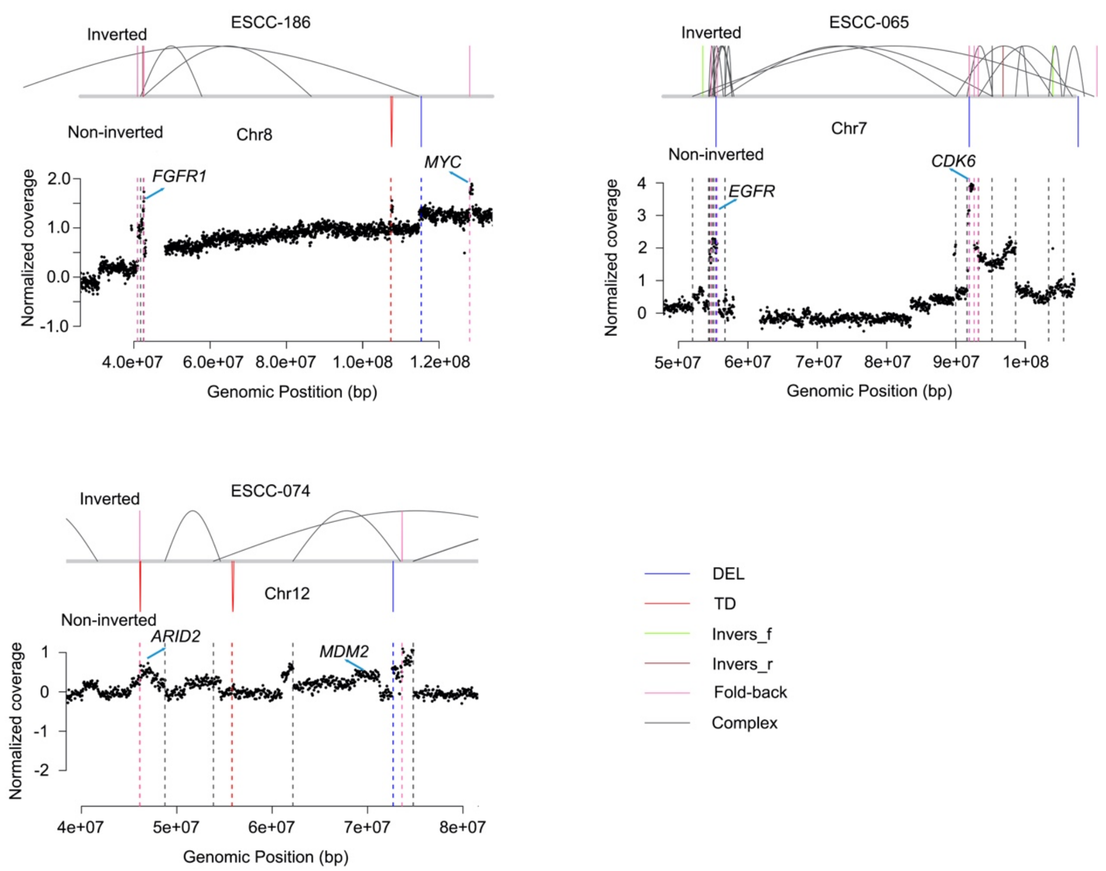

Supplementary Figure 18. Fold-back inversions amplified oncogenes together. Examples show SVs and coverage**,** *FGFR1* and *MYC* on chromosome 8, *EGFR* and *CDK6* on chromosome 7, and *ARID2* and *MDM2* on chromosome 12. Source data are provided as a Source Data file.

Supplementary Figure 19

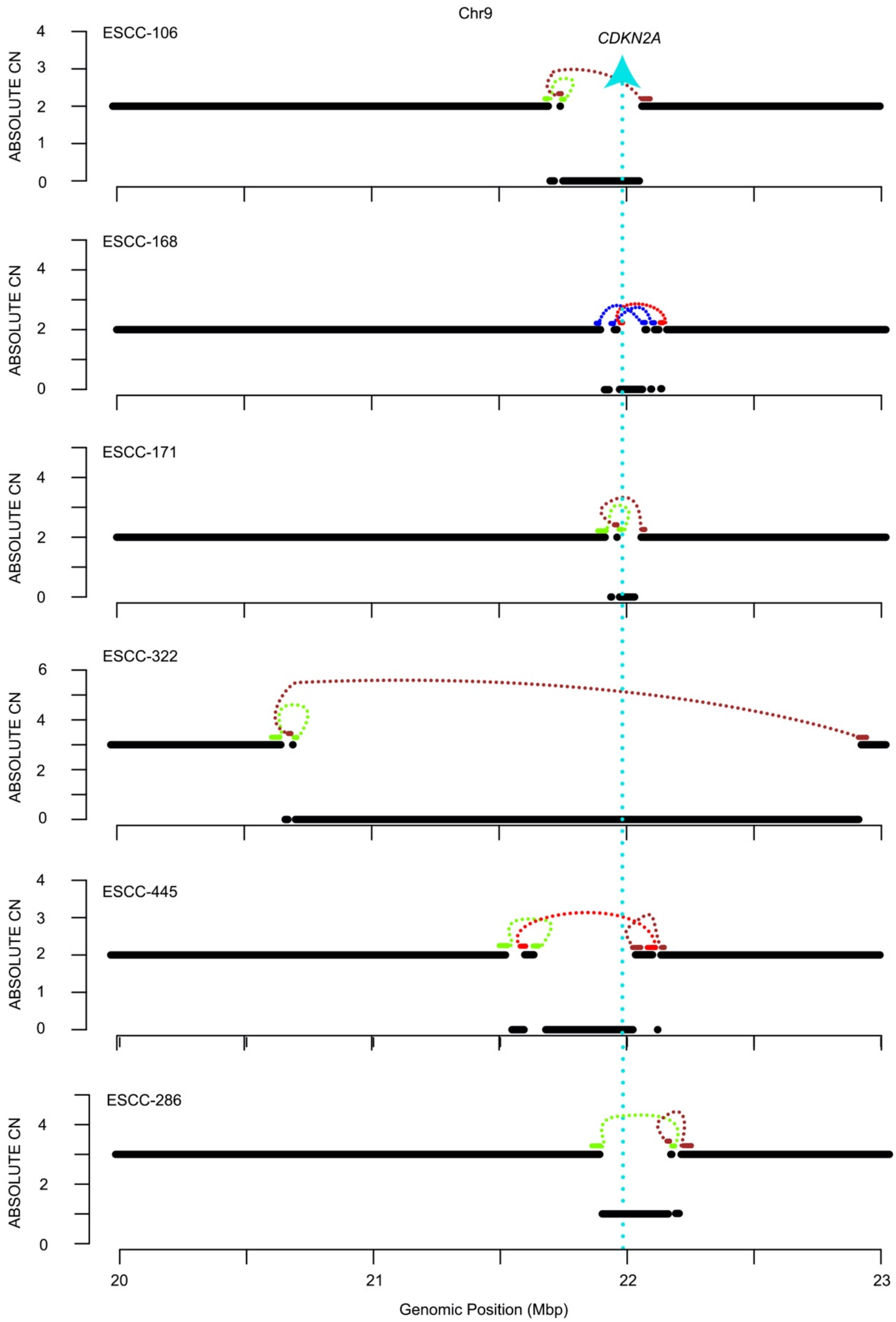

Supplementary Figure 19. *CDKN2A* deletions owing to unbalanced inversions in six ESCCs.

The x-axis represents the normalized coverage and y-axis show the absolute copy number. Source data are provided as a Source Data file.

Supplementary Figure 20

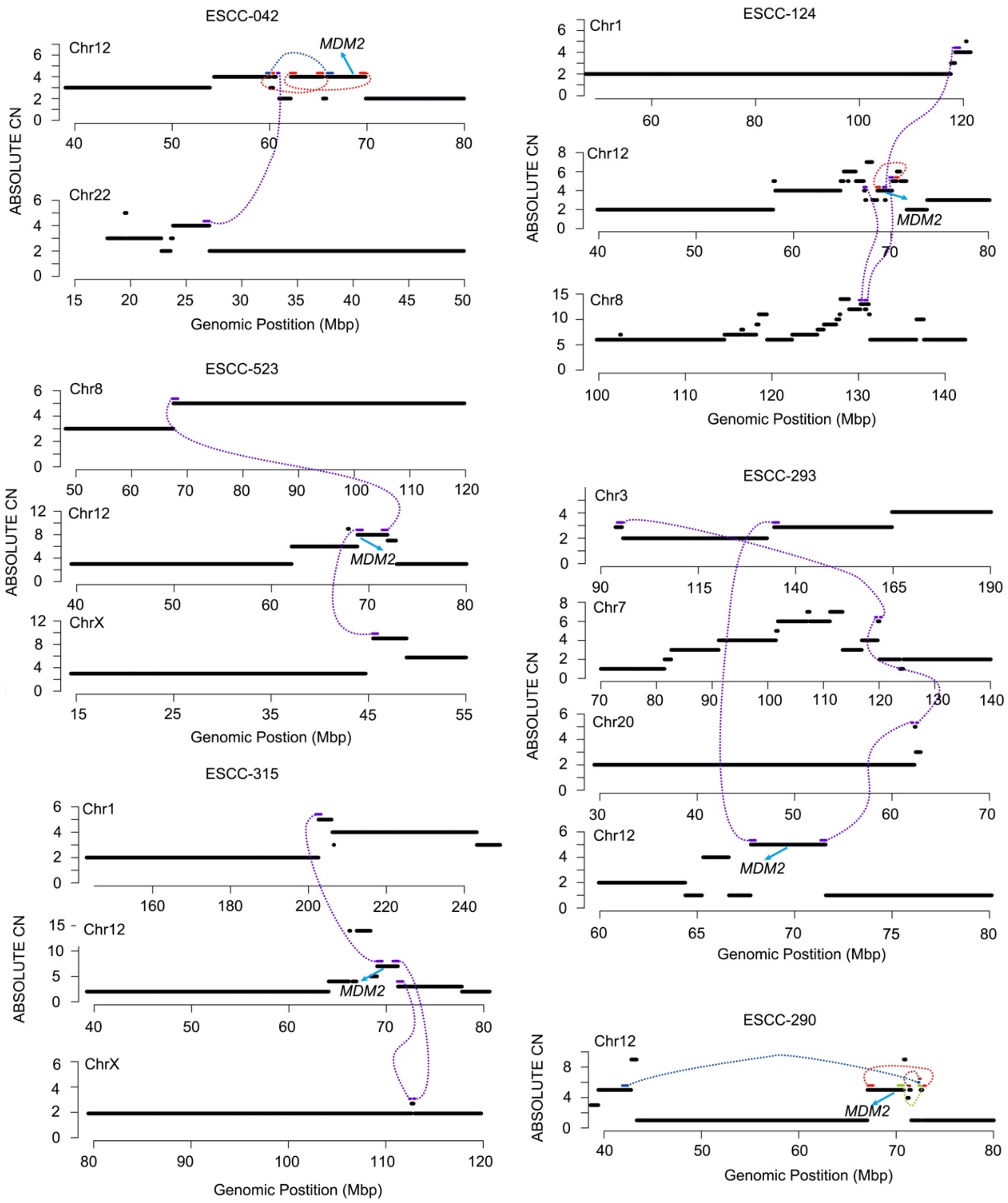

Supplementary Figure 20. *MDM2* amplifications derived from complex SV events. The black solid lines represent the copy number states of the target region, whereas structural variations are shown as colored dotted lines (blue: breakpoint strand "+-"; red: breakpoint strand "-+"; brown: breakpoint strand "--"; green: breakpoint strand "++") linking two segments. Source data are provided as a Source Data file.

Supplementary Figure 21

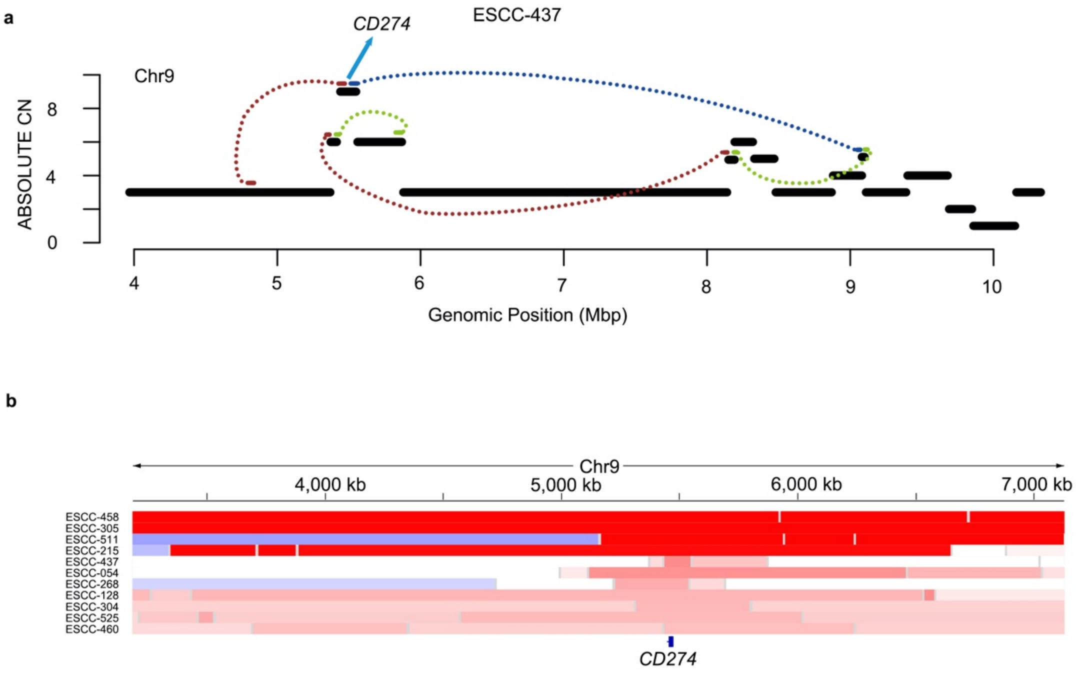

Supplementary Figure 21. Focal amplification of *CD274*. (a) *CD274* amplification derived from complex SV event. The black solid lines represent the copy number states of the target region, whereas structural variations are shown as colored dotted lines (blue: breakpoint strand "+-"; brown: breakpoint strand "--"; green: breakpoint strand "++") linking two segments. **(b)** The IGV heatmap of normalized coverage for amplifications of *CD274* in 10 ESCCs. The blue represents the deletion and red represent amplification. Source data are provided as a Source Data file.

Supplementary Figure 22

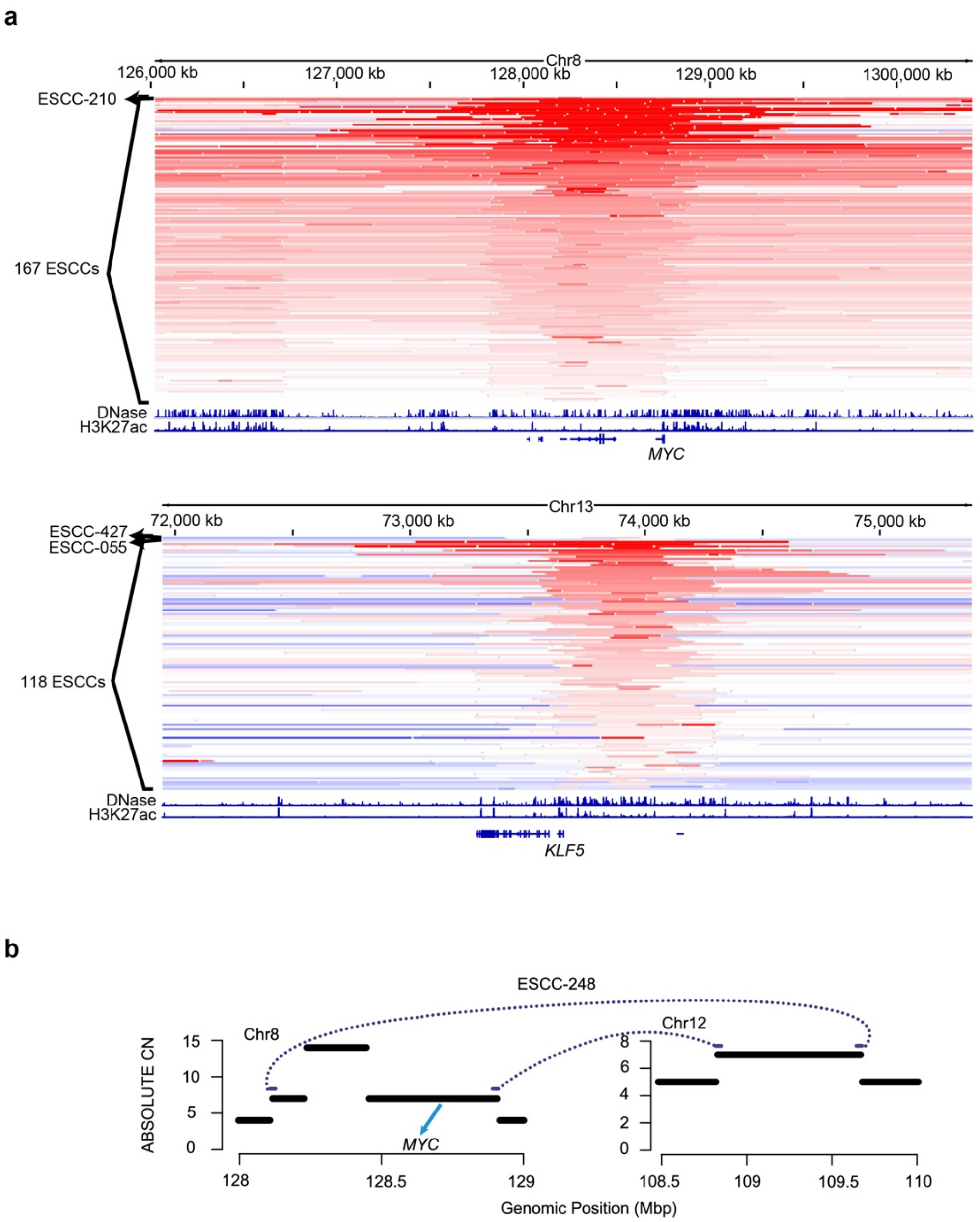

Supplementary Figure 22. SVs involve the regulatory regions of driver genes: MYC and KLF5. **(a)** CNV heat-maps show amplifications of *KLF5* super-enhancer and *MYC* super-enhancer. DNase and H3K27ac data from one sample EC074 in RoadMap are shown. **(b)** Examples: Amplifications of *MYC* super-enhancer owing to cycles of TD. Source data are provided as a Source Data file.

Supplementray Figure 23

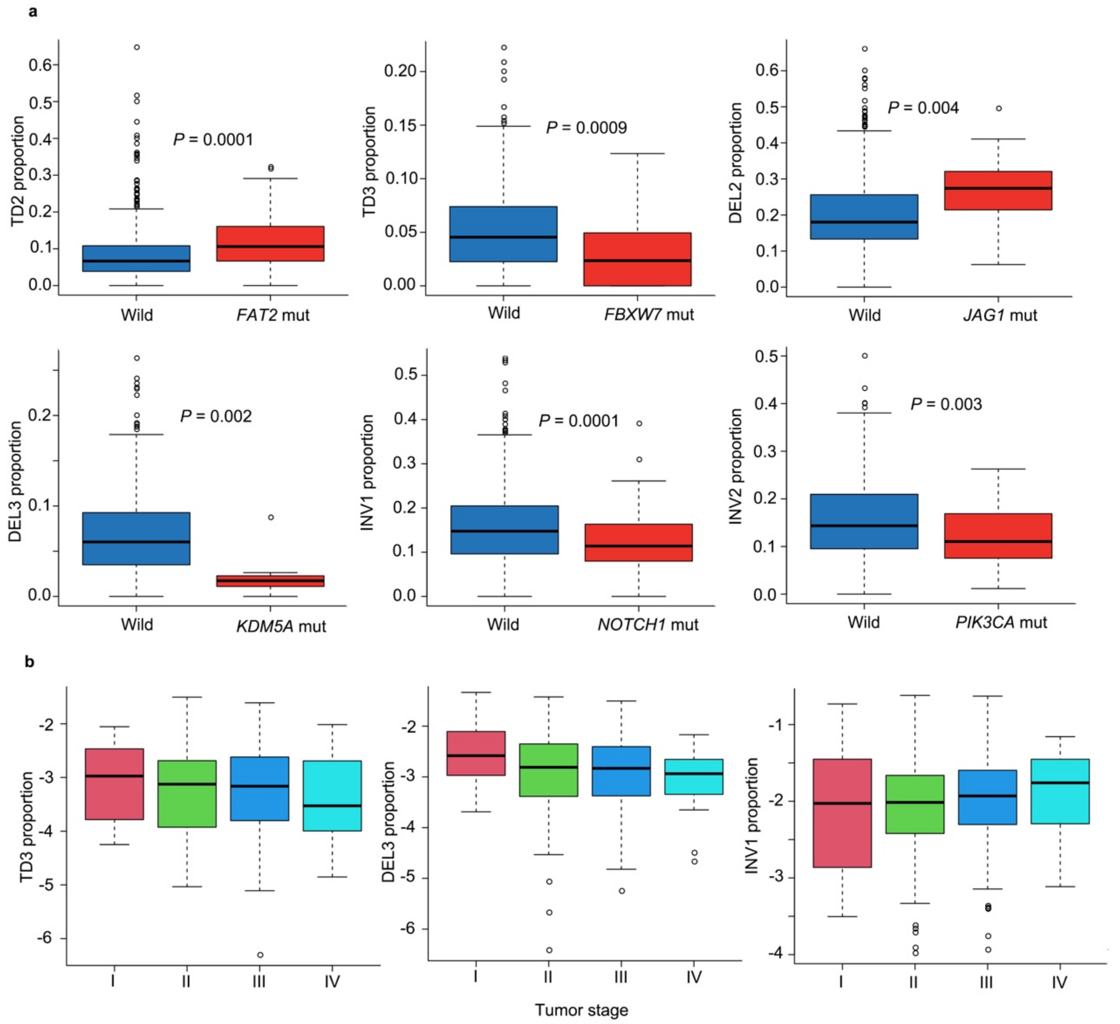

Supplementary Figure 23. The correlation of distinct SV signatures with driver mutations and Tumor stage. (a) Box plot of the correction between SV signatures and driver mutations. **(b)** Box plot of the correction between SV signatures and tumor stage. On the boxplots the horizontal line indicates the median, the box indicates the first to third quartile and whiskers indicate 1.5 × the interquartile range. Statistical analysis is performed with student-t test. n=528 biologically independent pairs of samples. Source data are provided as a Source Data file.

Supplementary Figure 24

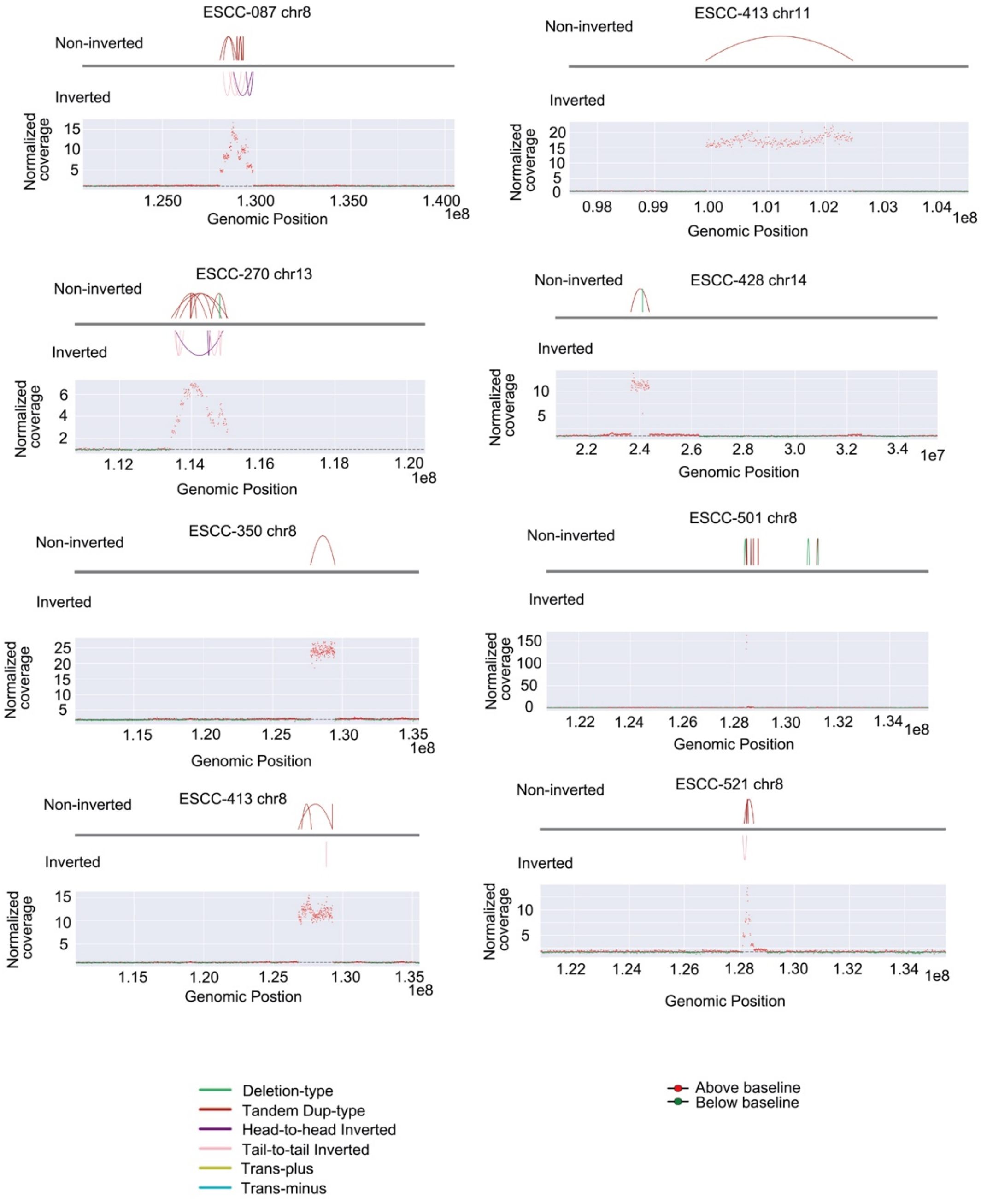

Supplementary Figure 24. Normalized coverage and SV events for 8 ecDNA events. Source data are provided as a Source Data file.

Supplementary Figure 25

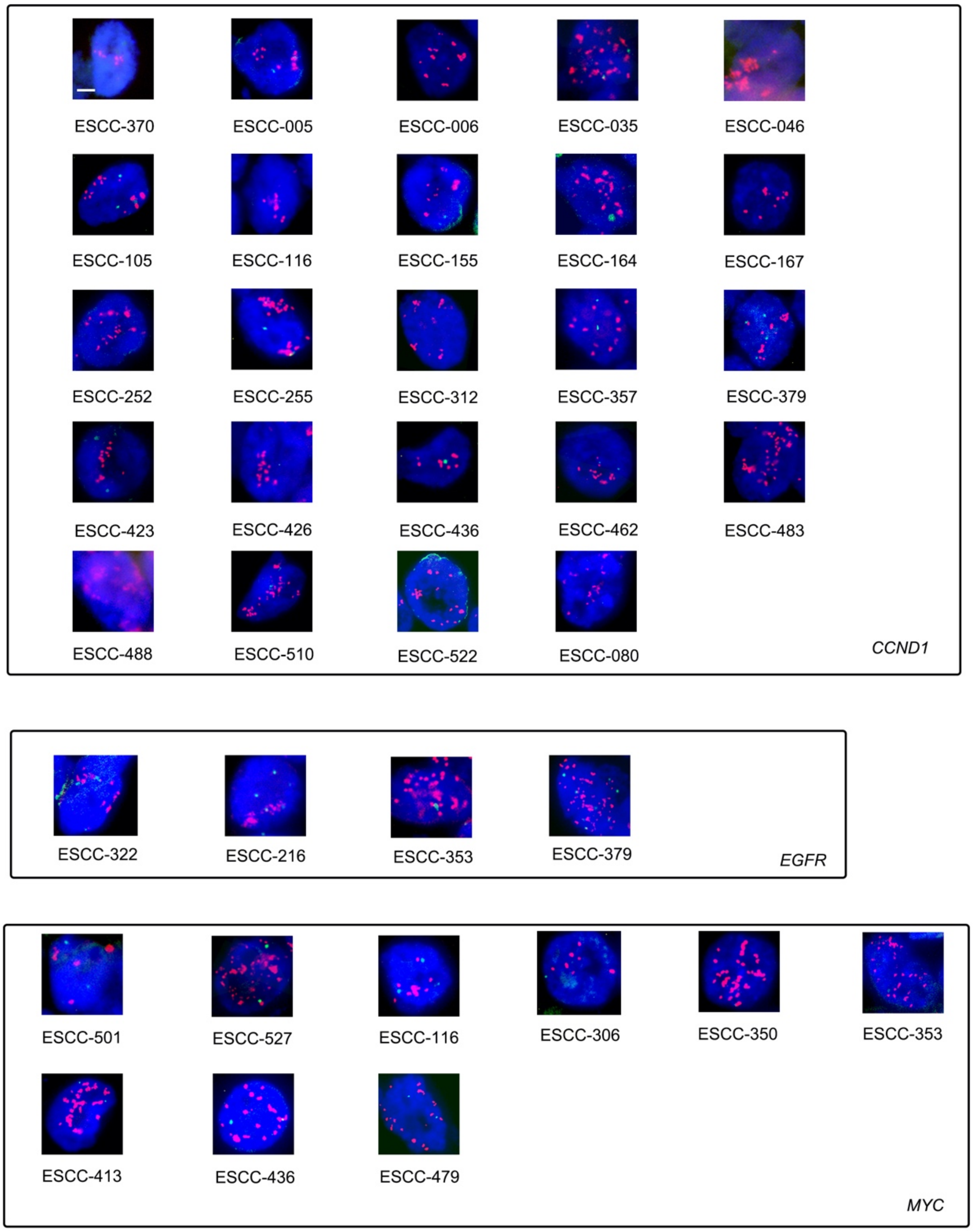

Supplementary Figure 25. Fluorescence in situ hybridization confirms ecDNA by showing gene amplification and its position as scattered multiple green signals in cells. Each part represents a gene and each small graph represents a tumor sample. Chromosome (red signal) and target gene (green signal) . Scale bars: 5 μm. Source data are provided as a Source Data file.

Supplementary Figure 26

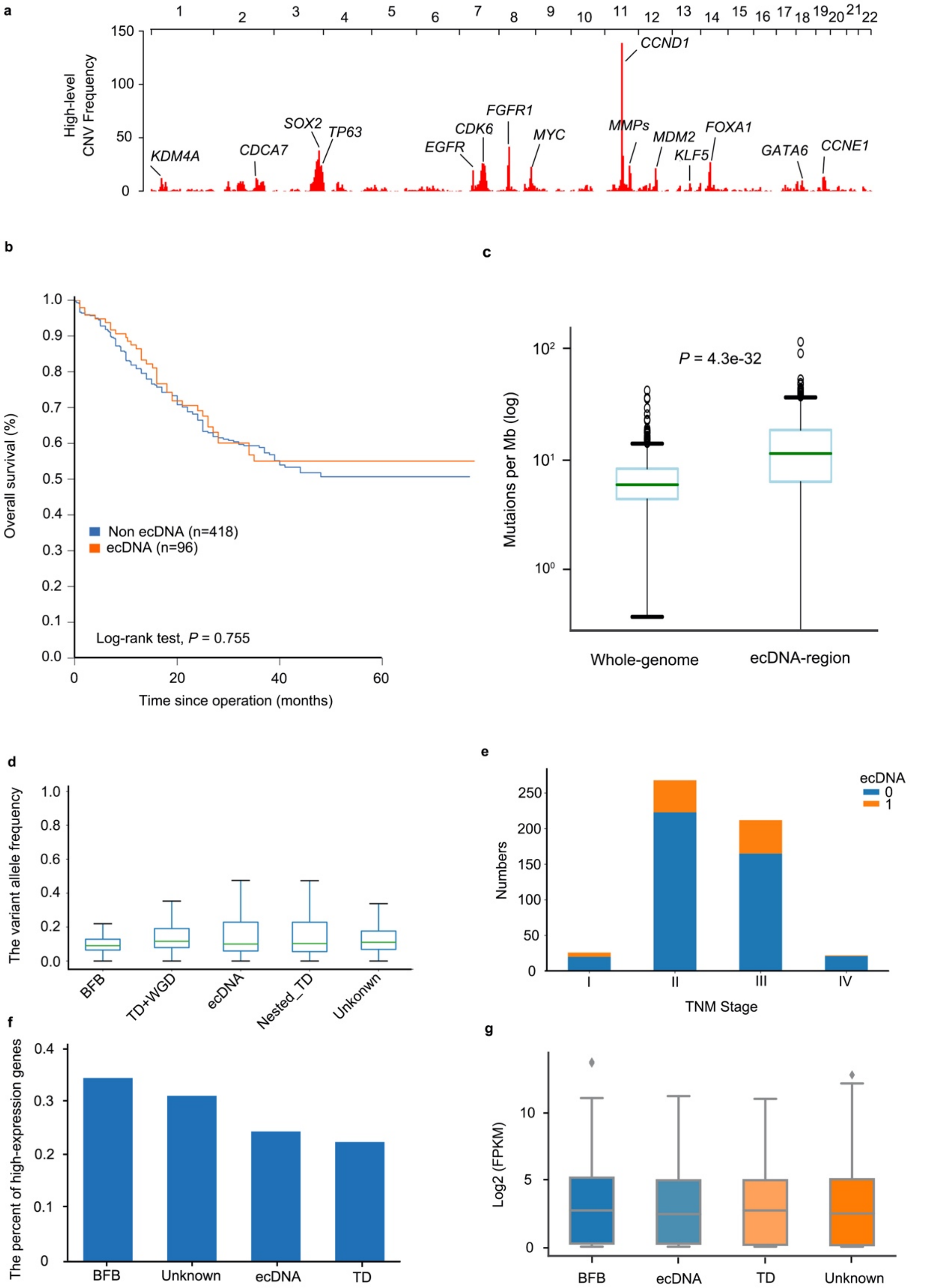

Supplementary Figure 26. Underlying mechanisms for amplicons. (a) The driver genes that are frequently amplified by amplicons. **(b)** The survival analyses of ecDNA in ESCCs. Statistical

analysis is performed with Log rank test. **(c)** Box plot shows the mutation rate of the genomic region. n=475 biologically independent pairs of samples. Statistical analysis is performed with student-t test. **(d)** Box plot shows that the variant allele frequency of somatic mutations involved in each mechanism. n=475 biologically independent pairs of samples. On the boxplots the horizontal line indicates the median, the box indicates the first to third quartile and whiskers indicate 1.5 × the interquartile range. **(e)** Bar plot shows the ecDNA distribution across tumor stages. **(f)** Bar plot shows the percent of high-expression genes in each mechanism. **(g)** Box plot shows the expression in each mechanism. On the boxplots the horizontal line indicates the median, the box indicates the first to third quartile and whiskers indicate 1.5 × the interquartile range. n=4072 genes. Source data are provided as a Source Data file.

Supplementary Table 1: Summary of hotspots identified for TDs.

TD-c1

chr	start.bp	end.bp	length.bp	number.bps	no.samples	Affect_gene	pvalue	rate.factor	hotspot.id
2	141433743	142389007	955264	94	44	LRP1B	0	15.27283	2_141.4Mb
3	188398518	189954900	1556382	62	28	TP63	0	6.717846	3_188.4Mb
11	65202690	71074333	5871643	137	54	CCND1	0	4.124075	11_65.2Mb
13	73586562	74234810	648248	38	19	KLF5	0	10.46444	13_73.6Mb

TD-c2

chr	start.bp	end.bp	length.bp	number.bps	no.samples	Affect.gene	pvalue	rate.factor	hotspot.id
1	209324934	209731201	406267	39	19	CD34	0	24.65771	1_209.3Mb
3	188849186	189923816	1074630	182	66	TP63	0	31.79881	3_188.8Mb
8	127030091	129061801	2031710	196	64	MYC	0	15.55084	8_127Mb
11	66639032	71580744	4941712	143	51	CCND1	0	5.701117	11_66.6Mb
11	100903468	103257668	2354200	43	19	MMPs	8.35E-13	3.598541	11_100.9Mb
12	27862840	28523752	660912	38	19	PTHLH	0	12.18575	12_27.9Mb
13	73512270	74386567	874297	205	80	KLF5	0	44.15223	13_73.5Mb
14	68510272	69943194	1432922	39	21	ZFP36L1	2.18E-13	4.05844	14_68.5Mb
20	10376981	11596441	1219460	67	26	JAG1	0	9.759758	20_10.4Mb

Supplementary Table 2: Complex rearangements tool

Complex rearrangements software	Paper	Complex rearrangements type
FindRear (tool available in github)	Our own paper	Cheomothripsis, Bridge deletion, Unbalanced inversion, TSI-mediated unbl-trams, Cycles of TSIs and TSI fold-back inversion.
gridss (tool available in github)	PMID: 34253237	BFB, ecDNA, rearrangements caused by SINE Alu, LINE L1HS insertions , or involving centromeric sequence.
Starfish (tool available in github)	PMID: 35835961	ecDNA,BFB, Large loss, Micronuclei, Large gain and Hourglass
SV clustering and annotation (not available)	PMID: 32025012	Chromoplexy, Cheomothripsis, Local-distant cluster, Local n-jump and Cycle of templated insertions

Supplementary Table 3: Primers used in this study

Enhancers in the front of PTHLH and Primers for cloning individual enhancers

Constituent Enhancer	Position	Forward primer	Reverse primer
e1	chr12: 28128000-28129000	CGGGGTACCCCG GCCCGGAGCGGTATTATTGA	CCGCTCGAGCGG ACGTCCAGCCTCGTGTCG
e2	chr12: 28134601-28135600	CGGGGTACCCCG AGGATTACTGGTATATGA	CCGCTCGAGCGG ACTACCCTTGGCATTCTGCCA
e3	chr12: 28176001-28177500	CGGGGTACCCCG TCTGAAAGCTTACAATCG	CCGCTCGAGCGG GCAGAAAGACTTCGTGGT
e4	chr12: 28178500-28181500	CGGGGTACCCCG GAGCTTGCAGTCTAGGGGCAG	CCGCTCGAGCGG AGAGCCTCGTTCTAGGGCCAG
e5	chr12: 28185600-28186900	CGGGGTACCCCG TCTGAAAGGCTAGTACACTC	CCGCTCGAGCGG AGGATGCTCT CCATCATTTA
e6	chr12: 28203501-28205500	CGGGGTACCCCG CAGAGCCGTGAGTCCTCTTTG	CCGCTCGAGCGG TTCATCTGTAAAGTGGTGATG
e7	chr12: 28283501-28284500	CGGGGTACCCCG GGAAACTGAGGCACAGAGTAGC	CCGCTCGAGCGG CCTTATATTTACATAGCAGGAC
e8	chr12: 28284501-28285500	CGGGGTACCCCG TATTCAGAGGTGGGTGGAGAAAC	CCGCTCGAGCGG AATGCAAGATAGGGTAGTATTTTC

Primer used for Realtime-PCR

Gene	Forward primer	Reverse primer
PTHLH	CTGGTTCAGCAGTGGAGCGT	AGGAAGAATCGTCGCCGTA
GAPDH	CGGAGTCAACGGATTTGGTCGTAT	AGCCTTCTCCATGGTGGTGAAGAC

Primers of sanger sequencing results of TDs in 11 samples.

Constituent	Forward primer	Reverse primer
ESCC-032	5'-GAGAAATTTTGCCTAATCCAAC-3'	5'-CCTAGCCAACAAATAATAGAGC-3'
ESCC-042	5'-GAAGCCATCACAAGAACAAC-3'	5'-GGTTGATTTTAATGAATGCCTA-3'
ESCC-167	5'-AATAATTCTTCAAATCCGA-3'	5'-TTTGCCATTTAATGTTACCTT-3'
ESCC-053	5'-TATCCTCTCCAGCATCTGT-3'	5'-GCCGACCAGGTTACACACTA-3'
ESCC-392	5'-CCAGAATATTGTCTTATGTGCTT-3'	5'-TTACCCTGCAAGTTAGTCAG-3'
ESCC-408	5'-TCTCCACCCTCAGCATCT-3'	5'-TACAAAAGGTTTAACTTGCG-3'
ESCC-329	5'-TTTAGCCCCTACCTACTCA-3'	5'-CACCCATCTTCCAATCCA-3'
ESCC-381	5'-ATCAGAGCGAATAACAAGCA-3'	5'-TTCCCAACCCAACATCAG-3'
ESCC-006	5'-GGGGACACAGATTCAAACCAT-3'	5'-TCACTCCCCATTATCCCCTT-3'
ESCC-461	5'-AGAGGAGGAGGAGGAGGAAG-3'	5'-CAGTTTCACTCTGTTGCCCA-3'
ESCC-191	5'-GTCATATCTCCTTAGGCTG-3'	5'-CCTAATTCCAAACACCGTA-3'

Supplementary Table 4. Primers used for plasmid construction, Realtime-PCR and Cas9

sg RNA

sg RNA for e3	Forward: TTGAACATGACAGTCCGCTT	Reverse: CTTACTGGGGGTCTATGGTC
	Forward: TGGTCACCTGGGGAATTATA	Reverse: AAAAGGTCTTCACCCTAGAC
sg RNA for e5	Forward: GTCTATATATTAGAGCGCTG	Reverse: ACTAATAGCCCAATTACTAG
	Forward: GGTGAGAAGCACACATGCTA	Reverse: ACTGTGGTAGCTCTACAAC

Primer used for detecting the effect of cas9

e3	Outside forward: TGCAAAGCCTAACTTACACGATC	Outside reverse: CAGGCCATAGGATGAGCTCTCG
	Inside forward:TTACATGGCCTCATCTCCCATAGC	Inside reverse: TGAGTGGAAATAGGTGCTCCATG
e5	Outside forward: ATGCTGAGGCTTATATAAGAAT	Outside reverse: AGTTCGCCAAGGACTTAGCCAG
	Inside forward: TTGTGTAGTCTGTAACTGGGT	Inside reverse: ACTTACTGTTGAAGTCATCCATG
