# Supplementary material for: Characterization of somatic structural variations in 528 Chinese individuals with Esophageal squamous cell carcinoma
Source: Nat Commun. 2022 Oct 22;13:6296. doi: 10.1038/s41467-022-33994-3 (PMC9588063; doi:10.1038/s41467-022-33994-3)
Supplement: Supplementary file 2 — Description of Additional Supplementary Files [file 41467_2022_33994_MOESM2_ESM.pdf]

1. File Name: Supplementary Data 1

   Description: Clinical information and features

2. File Name: Supplementary Data 2

   Description: Total SVs events

3. File Name: Supplementary Data 3

   Description: Total Complex rearrangements, including Bridge deletion, Circular TD and Unbalanced inversion.

4. File Name: Supplementary Data 4

   Description: Total chromothrisis events

5. File Name: Supplementary Data 5

   Description: 185 DEGs used for the classifier to detect ESCC with TSI fold-back

6. File Name: Supplementary Data 6

   Description: Identified simple and TSI fold-back inversions.

7. File Name: Supplementary Data 7

   Description: High-level amplification events of ESCC.
