# Supplementary material for: Characterization of somatic structural variations in 528 Chinese individuals with Esophageal squamous cell carcinoma
Source: Nat Commun. 2022 Oct 22;13:6296. doi: 10.1038/s41467-022-33994-3 (PMC9588063; doi:10.1038/s41467-022-33994-3)
Supplement: Supplementary file 10 — Reporting Summary [file 41467_2022_33994_MOESM10_ESM.pdf]

nature portfolio

Corresponding author(s): Yongping Cui, Zhihua Liu, Qimin Zhan

Last updated by author(s): Oct 4, 2022

# Reporting Summary

Nature Portfolio wishes to improve the reproducibility of the work that we publish. This form provides structure for consistency and transparency in reporting. For further information on Nature Portfolio policies, see our Editorial Policies and the Editorial Policy Checklist.

## Statistics

For all statistical analyses, confirm that the following items are present in the figure legend, table legend, main text, or Methods section.

| n/a | Confirmed | |
|---|---|---|
| ☐ | ☒ | The exact sample size (*n*) for each experimental group/condition, given as a discrete number and unit of measurement |
| ☐ | ☒ | A statement on whether measurements were taken from distinct samples or whether the same sample was measured repeatedly |
| ☐ | ☒ | The statistical test(s) used AND whether they are one- or two-sided<br>*Only common tests should be described solely by name; describe more complex techniques in the Methods section.* |
| ☐ | ☒ | A description of all covariates tested |
| ☐ | ☒ | A description of any assumptions or corrections, such as tests of normality and adjustment for multiple comparisons |
| ☐ | ☒ | A full description of the statistical parameters including central tendency (e.g. means) or other basic estimates (e.g. regression coefficient) AND variation (e.g. standard deviation) or associated estimates of uncertainty (e.g. confidence intervals) |
| ☐ | ☒ | For null hypothesis testing, the test statistic (e.g. *F*, *t*, *r*) with confidence intervals, effect sizes, degrees of freedom and *P* value noted<br>*Give P values as exact values whenever suitable.* |
| ☒ | ☐ | For Bayesian analysis, information on the choice of priors and Markov chain Monte Carlo settings |
| ☒ | ☐ | For hierarchical and complex designs, identification of the appropriate level for tests and full reporting of outcomes |
| ☐ | ☒ | Estimates of effect sizes (e.g. Cohen's *d*, Pearson's *r*), indicating how they were calculated |

*Our web collection on statistics for biologists contains articles on many of the points above.*

## Software and code

Policy information about availability of computer code

| | |
|---|---|
| Data collection | No software was used to collect data. |
| Data analysis | All softwares used are described in the methods section. Publicly available softwares for Next-generation Sequencing analysis:Skewer (v0.2.2), STAR(v2.4.2a), RSEM (1.2.29),GATK4, MutTect2, Platypus(0.8.1), SvABA(1.1.3), Delly(0.9.1), MutationalPatterns(3.2.0), Patchwork(1.0), ABSOLUTE(1.0), SeqKat(0.0.8), AmpliconArchitect(1.2), NanoPack, Minimap2, . The complex SVs were defined by FindRear and the source code has uploaded to github (https://github.com/ZHOUYong0530/FindRear). All statistics analysis and survival analysis were performed in R (version 4.0). |

For manuscripts utilizing custom algorithms or software that are central to the research but not yet described in published literature, software must be made available to editors and reviewers. We strongly encourage code deposition in a community repository (e.g. GitHub). See the Nature Portfolio guidelines for submitting code & software for further information.

## Data

Policy information about availability of data

All manuscripts must include a data availability statement. This statement should provide the following information, where applicable:

- Accession codes, unique identifiers, or web links for publicly available datasets
- A description of any restrictions on data availability
- For clinical datasets or third party data, please ensure that the statement adheres to our policy

The raw sequencing data generated in this study have been deposited in the Genome Sequence Archive (Genomics, Proteomics & Bioinformatics 2021) in National Genomics Data Center (Nucleic Acids Res 2022), China National Center for Bioinformation / Beijing Institute of Genomics, Chinese Academy of Sciences (GSA-Human: HRA003107 (WGS&RNA-seq, https://ngdc.cncb.ac.cn/gsa-human/browse/HRA003107), HRA000021 (WGS, https://ngdc.cncb.ac.cn/gsa-human/browse/

HRA000021) and HRA002508 (WGS & Nanopore, https://ngdc.cncb.ac.cn/gsa-human/browse/HRA002508)). The raw sequencing data are available under controlled access due to data privacy laws related to patient consent for data sharing and the data should be used for research purposes only. Access can be obtained by approval via their respective DAC (Data Access Committees) in the GSA-human database. According to the guidelines of GSA-human, all non-profit researchers are allowed access to the data and the Principle Investigator of any research group is allowed to apply for Controlled-access of the data. For data requests, please refer to the detailed guide: https://ngdc.cncb.ac.cn/gsa-human/document/GSA-Human_Request_Guide_for_Users_us.pdf. DAC will respond within two weeks. The data will be available within a week once the access has been granted and they will be available to download for one year. The human genome database used in this paper is version hg19 (https://hgdownload.soe.ucsc.edu/goldenPath/hg19/bigZips/). The publicly available RNA-seq data was downloaded from GEO database with accession number GSE53625[31] (https://www.ncbi.nlm.nih.gov/geo/query/acc.cgi?acc=GSE53625). The publicly available Chip-seq data used in this study are available in the GEO database under accession code GSE155187[26] (https://www.ncbi.nlm.nih.gov/geo/query/acc.cgi?acc=GSE155187). Source data are provided in this paper as a Source Data file. The remaining data are available within the Article, Supplementary Information or Source Data file.

# Field-specific reporting

Please select the one below that is the best fit for your research. If you are not sure, read the appropriate sections before making your selection.

[x] Life sciences   [ ] Behavioural & social sciences   [ ] Ecological, evolutionary & environmental sciences

For a reference copy of the document with all sections, see nature.com/documents/nr-reporting-summary-flat.pdf

# Life sciences study design

All studies must disclose on these points even when the disclosure is negative.

| | |
|---|---|
| Sample size | We performed whole-genome sequencing on regional tumor samples and adjacent normal tissues from 528 ESCC patients, of which 133 pairs also were sequenced by RNA-seq. In addition, We supplemented the WGS and Nanopore sequencing of two ESCC samples. |
| Data exclusions | The ESCC regional tumor samples produced low quality sequencing data were excluded. |
| Replication | For all experiments, at least three independent experiments were performed and each experiment was performed in triplicate. All results of duplicates were consistent. |
| Randomization | The ESCC patients were collected randomly to form the cohort. All the studies ESCC patients were retrospective and did not need randomly grouping. And cells were allocated into experimental groups randomly. |
| Blinding | Investigators were blinded to the group allocation during cell implantation or sample/data collection. |

# Reporting for specific materials, systems and methods

We require information from authors about some types of materials, experimental systems and methods used in many studies. Here, indicate whether each material, system or method listed is relevant to your study. If you are not sure if a list item applies to your research, read the appropriate section before selecting a response.

## Materials & experimental systems

| n/a | Involved in the study | |
|---|---|---|
| [ ] | [x] | Antibodies |
| [ ] | [x] | Eukaryotic cell lines |
| [x] | [ ] | Palaeontology and archaeology |
| [x] | [ ] | Animals and other organisms |
| [x] | [ ] | Human research participants |
| [x] | [ ] | Clinical data |
| [x] | [ ] | Dual use research of concern |

## Methods

| n/a | Involved in the study | |
|---|---|---|
| [x] | [ ] | ChIP-seq |
| [x] | [ ] | Flow cytometry |
| [x] | [ ] | MRI-based neuroimaging |

## Antibodies

| | |
|---|---|
| Antibodies used | PTHLH antibody (ab197358, Abcam, Cambridge, UK) (1:200 dilution) was used as the primary antibody in immunohistochemistry. |
| Validation | Antibodies used IHC were validated using negative and positive controls and underwent an optimization process including titration, variation of antigen retrieval process, and incubation periods, and further testing in a select set of clinical samples before they were applied to a large scale cohorts. |

## Eukaryotic cell lines

Policy information about cell lines

| | |
|---|---|
| Cell line source(s) | ESCC cell lines KYSE180, KYSE150 and KYSE450 cell line were purchased from Cell Bank of Type Culture Collection of Chinese |

| | |
|---|---|
| | Academy of Sciences. |
| Authentication | All of the cells were authenticated by short tandem repeat (STR) analysis. |
| Mycoplasma contamination | All cell lines were routinely tested to ensure they are free of mycoplasma contamination (VenorTMGeM Mycoplasma Detection Kit, Sigma-Aldrich). |
| Commonly misidentified lines (See ICLAC register) | No commonly misidentified cell lines were used. |
